# Growing attention of immunogenicity among patients with autoimmune diseases post-SARS-CoV-2 vaccination: meta-analysis and systematic reviews of the current studies

**DOI:** 10.1080/07853890.2025.2478319

**Published:** 2025-03-26

**Authors:** Chao Zhang, Yu-Qiang Zhang, Run-Ben Liu, Yu-Tong Ma, Lin-Kang Zhao, Fu-Qiang Yin, Jing Tu, Yang-Yang Yao

**Affiliations:** ^a^ Center for Evidence-Based Medicine and Clinical Research, Hubei Provincial Clinical Research Center of Central Nervous System Repair and Functional Reconstruction, Taihe Hospital, Hubei University of Medicine, Shiyan, China; ^b^ Department of Neurosurgery, Hubei Provincial Clinical Research Center of Central Nervous System Repair and Functional Reconstruction, Taihe Hospital, Hubei University of Medicine, Shiyan, China; ^c^ Department of Anesthesiology and Perioperative Medicine, The Second Affiliated Hospital and Yuying Children’s Hospital of Wenzhou Medical University, Wenzhou, Zhejiang, China

**Keywords:** Autoimmune disease, immunogenicity, SARS-CoV-2 vaccines, meta-analysis

## Abstract

**Objective:**

This study aimed to identify the optimal strategy for patients with autoimmune diseases by comparing the immunoreaction and effectiveness of severe acute respiratory syndrome coronavirus 2 (SARS-CoV-2) vaccines between healthy individuals and patients.

**Methods:**

The PubMed, Embase, and Cochrane Library were searched for eligible studies on effectiveness and immunoreaction to SARS-CoV-2 vaccines in patients with autoimmune diseases published until October 07, 2022. The quality of each included study was evaluated by independent reviewers using National Institutes of Health study quality assessment tool, and the STATA 15.0 software was used for all statistical analyses.

**Results:**

A total of 84 publications were included and analyzed in this meta-analysis, favoring healthy controls regarding serological response (risk ratio, RR=0.88, 95% CI (confidence interval): 0.86–0.91), antibody response (RR=0.90, 95%CI: 0.87–0.94), and incidence of seropositive immunoglobulin G (IgG) (RR=0.74, 95%CI: 0.69–0.80) than patients post-vaccination. Patients with autoimmune diseases developed lower IgG (standard mean difference, SMD=−0.64 95%CI: −0.84 to −0.43) and antibody titer level (SMD=−1.39, 95%CI: −2.30 to −0.49) than healthy individuals in AU/ml. Stratified analyses were conducted further according to various potential factors in full-text studies.

**Conclusion:**

Patients who are immunocompromised and received more vaccines demonstrated poorer humoral responses and seropositive incidence after SARS-CoV-2 vaccination than healthy individuals. Despite the lack of observable favor for patients with autoimmune diseases, the trend of effectiveness of SARS-CoV-2 vaccines is close to that for healthy populations. Patients who are immunocompromised should be provided a better SARS-CoV-2 vaccination schedule, considering various vaccine subtypes, dose(s), variants of concern, and immunoassays.

## Introduction

Vaccines for severe acute respiratory syndrome coronavirus 2 (SARS-CoV-2) were developed during the emergence of SARS-CoV-2, and various vaccine types and subtypes had been produced in such short period as well [1]. Mainstream vaccine types including messenger RNA (mRNA) platforms, adenoviral vectors, and inactivated virus vaccines have been vaccinated in many countries and cities [2]. The widespread promotion of vaccine platforms significantly bolstered public confidence in acquiring immunological protection amid viral spread. However, vaccines have indefinite efficiency and safety in among large and complex populations worldwide, such as healthy populations and patients who are immunocompromised. One study has emphasized that type and composition of mRNA formulation was linked to difference between safety of BNT162b2 and mRNA-1273 vaccines [3]. Haidar et al. [4] have reported higher antibody level in patients with hematologic malignancy who received mRNA-1273 than those who received BNT162b2 after the third dose. Additionally, another study has indicated that patients who are immunocompromised do not exhibit a striking pattern of increase in immunogenicity following mRNA vaccine compared to healthy population [5]. Moreover, inactivated vaccines have a certain probability of exerting harmful effects in patients rather than providing protection from the virus [6]. Thus, this can lead to the hypothesis that distinct vaccine types and combination protocol patients applied could be potentially shaped the strong or weak immune response after immunizations. Therefore, the influence of various vaccine types on patients with autoimmune diseases including homologous and heterogeneous vaccine scheme needs to be explored. For patients who are immunocompromised, no unified definition scope exists. Moreover, unpredicted SARS-CoV-2 variants of concern might threaten the immunity of patients who are immunocompromised [7,8]. Since patients with autoimmune diseases take immunosuppressive drugs and receive biological therapy, referring to multiple administrations of treatments (e.g. anti-tumor necrosis factor (TNF), methotrexate, ustekinumab, secukinumab, and prednisone) with different diseases associated with efficiency of SARS-CoV-2 vaccines [8]. Several studies have reported on the considerable differences in the effectiveness between variants and proved that vaccine recipients have decreased effectiveness during emergence of the Delta variant, such as the B.1.617.2 variant [9,10]. The influence of different types of SARS-CoV-2 on the immunogenicity of patients who are immunocompromised is another potential point of interest. Overall, various strategies regarding SARS-CoV-2 vaccination have been proposed, and whether various efficiency of SARS-CoV-2 vaccines types, vaccine doses (first dose, second dose, third dose and even forth dose), history of previous SARS-CoV-2 infection, SARS-CoV-2 variants of concerns, utilized immunoassay, administration of immunosuppression treatments, and different kind of autoimmune disease patients diagnosed could possibly affect humoral response among those patients with autoimmune diseases is controversial.

Although several previous meta-analyses and systematic reviews have revealed that some patients with autoimmune inflammatory diseases (AIDs) receive more benefits of SARS-CoV-2 vaccines than harmful effects, sufficient data on these patients are also lacking [11–13]. A recent retrospective study [14] has highlighted that not all individuals who are immunocompromised have strong serological responses after receiving mRNA vaccines following the third dose with 6 months of follow-up. Nevertheless, no comprehensive and acquired meta-analysis was published on autoimmune disease (such as AIDs, immune mediated inflammatory diseases [IMIDs], autoimmune rheumatic diseases [ARDs], and inflammatory bowel disease [IBD]), except for patients who received organ transplantation, those with cancer, and those with acquired immune deficiency syndrome [AIDS]. Thus, we performed a meta-analysis and systematic reviews focused on the abovementioned concerns to explore the efficiency of mainstream SARS-CoV-2 vaccines on patients with autoimmune diseases in terms of outcomes including seroconversion rate; antibody response; antibody titer; seropositive incidence of immunoglobin G (IgG); serological IgG titer; seropositive incidence of neutralizing antibody (NAb), NAb inhibitor serological NAb, or NAb inhibitor titer; and breakthrough infection.

### Materials and methods

This meta-analysis was performed according to the Preferred Reporting Items for Systematic Reviews and Meta-Analyses (PRISMA) guidelines [15] to reveal flow of identifying eligible studies. No ethical approval was required for the study.

### Search strategy

The PubMed, Embase, and Cochrane Library databases were searched from inception until October 07, 2022 using the terms ‘COVID-19’, ‘COVID-19 virus’, ‘SARS-CoV-2’, ‘SARS2’, ‘Coronavirus Disease 2019’, ‘2019-nCoV’, ‘2019 Novel Coronavirus’, ‘SARS Coronavirus 2’, ‘vaccines’, ‘immunocompromised’, and ‘autoimmune diseases’ to obtain full texts and abstracts published in the English language. References in the included studies were manually searched to supplement the electronic search. Two reviewers independently evaluated all full-text manuscripts for eligibility. Any disagreements between them were resolved by a third reviewer.

### Eligibility criteria

The inclusion criteria for included articles were as follows: (1) the exposure group comprises patients with autoimmune diseases including ARDs, AIDs, IMIDs, IBDs, chronic inflammatory diseases (CIDs), and combined variable immune deficiency (CVID) who had been vaccinated with SARS-CoV-2 vaccines; (2) the non-exposure group was defined as the general population (i.e. healthy volunteers); and (3) studies expounding the outcomes involving seroconversion rate, antibody response, seropositive incidence of IgG, antibody (SARS-CoV-2) titer, NAb or NAb inhibition titer, and breakthrough infection.

The exclusion criteria were as follows: (1) studies involving patients with hematological malignancy or solid cancer, AIDS or HIV virus, and organs transplant; (2) studies including children or pediatric patients aged <12 years; and (3) publications in languages other than English.

### Quality assessment

Regarding SARS-CoV-2 vaccination trials on patients with autoimmune diseases, randomized controlled trials have not been conducted yet. Thus, the NIH study quality assessment tool [16] was used to measure the risk of bias in case–control and observational cohort studies. This assessment tool comprises 14 items for observational cohort studies and 12 items for case–control studies. All items could be answered as ‘yes’, ‘no’, ‘not reported’, or ‘not applicable’, and the overall quality of each study could be classified as ‘good’, ‘fair’, or ‘poor’.

### Data extraction and outcome measures

Two reviewers extracted relevant data from each literature, involving key information of eligible literature, that is, the basic information, primary outcomes on the effectiveness of SARS-CoV-2 vaccines. Specifically, information on study characteristics (author, year, study design, sample, and country), participant information (gender, age, therapy regimens, particular diseases, and immunogenicity analysis tools or immunoassay) and SARS-CoV-2 vaccination schemes (vaccine type or subtype, vaccine doses, and seropositive cutoff value) was collected. Literature screening was completed by the two reviewers individually, and any discrepancies were resolved by a third researcher.

We included all eligible studies reporting on the effectiveness of SARS-CoV-2 vaccines in patients with autoimmune diseases. The primary outcomes of this study were the seroconversion rate and SARS-CoV-2 antibody titer in patients who are immunocompromised. The secondary outcomes included antibody response, seropositive incidence of IgG, IgG titer, breakthrough infection, seropositive incidence of NAb or NAb inhibitor, and NAb or NAb titer.

### Statistical analysis

The effect size of dichotomous data is presented as risk ratios (RRs) and was calculated using the Mantel–Haenszel method with a corresponding 95% confidence interval (CI). Continuous data, such as IgG titer, are reported as standard mean differences (SMDs) with 95%CI to present the efficiency of SARS-CoV-2 vaccine regimens. Moreover, heterogeneity among original articles was appraised using the Higgins’s I^2^ test. When I^2^ > 40%, a random effect model was used. Otherwise, a fixed-effects model was used. A subgroup analysis of setting outcomes was performed to evaluate studies based on the vaccine doses, vaccine subtype (BNT162b2, mRNA-1273, AD26.COV2.S and AZD1222, Sinovac-CoronaVac, Vero cell, and BBV152), and type (mRNA, inactivated virus, and adenoviral vector vaccines) received by the recruited people, number of vaccine injections (first, second, third, and fourth SARS-CoV-2 vaccinations), history of SARS-CoV-2 infection, SARS-CoV-2 variants of concern, immunoassay, particular diseases, and therapy regimens. The STATA 15.0 software was used for all subgroup analyses.

## Results

### Literature identification and characteristics of the included studies

**Figure 1.**
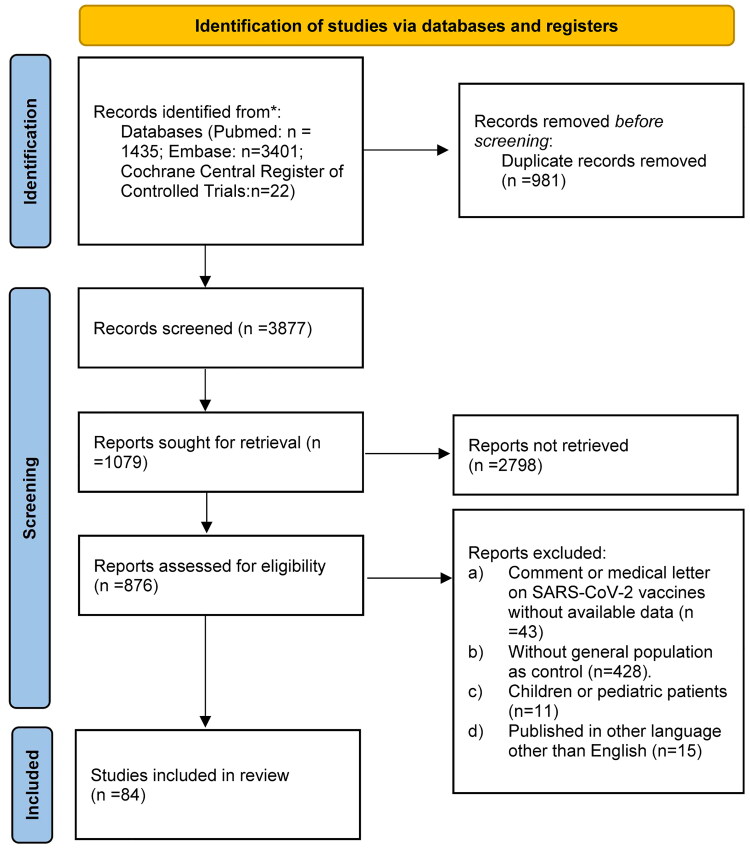
PRISMA 2020 flow diagram for new systematic reviews which included searches of databases and registers only.

In total, 4858 studies were obtained by searching digital databases. We then identified 1079 publications after applying the inclusion and exclusion criteria. Finally, 84 studies [17-100] were included in this meta-analysis and systematic review after full-text screening of 876 potential studies. Figure 1 presents the study inclusion flowchart. Nineteen studies were observational cohort studies, published online from 2020 to 2023, involving diverse populations with various autoimmune diseases. The most commonly received SARS-CoV-2 vaccine subtypes by the targeted patients and healthy volunteers were BNT162b2, mRNA-1273, AZD1222, Vero cell, AD16.COV2.S, and Sinovac-CoronaVac. Nearly all participants received at least one SARS-CoV-2 vaccine dose, and various cut-off values of the positive antibody titer criteria are reported in Table 1.

**Table 1.** The statistics of the included studies.

Study	Year	Study design	Sample	Country	Ethnicity	Gender	Age Mean (SD)	Previous history of SARS-CoV-2 infection
Alexander et al.	2022	Multicenter, prospective, case-control	481 IBD and 121 Healthy controls	UK	White, Non-White, Asian, Mixed, Black	Female/male: SLE:125/158; Healthy controls:56/30	42.3 (28.0,59.5)	108
Boekel et al.-1st	2021	multicenter, prospective, cohort	632 IMID and 289 control	Netherland	NA	Female:423	63 (11)	104
Boekel et al.-2nd	2021	multicenter, prospective, cohort	632 IMID and 289 control	Netherland	NA	Female:424	63 (11)	104
Deepak et al.	2021	Multicenter, prospective observational cohort	133 CID and 53 immunocompetent participates	USA	White, Asian, Black, or African American	Female:99; Immunocompetent participants:29	45.5 (16.0); immunocompetent patients:43.4 (14.1)	NR
Edelman-Klapper et al.	2022	Multicenter, prospective observational cohort	182 IBD and 73 Healthy controls	Israel	Ashkenazi, non-Ashkenazi	IBD: female:73; Healthy controls:53	IBD: 37.9 (14.3); Healthy controls: 36.6 (12.4)	NR
Ferri et al.	2021	Multicenter, prospective, observational	478 ASD and 502 Healthy controls	Italy	NA	ASD: Female/male: 401/77	ASD: 59 (15);	NR
Furer et al.	2021	Multicenter, prospective, observational	686 IRD and 121 controls	Israel	NA	Female: 475	Median: 59	NR
Geisen et al.	2021	Single-center, cohort	26 CID and 42 controls	Germany	NA	CID: Female: 17/26; Healthy controls:29/42	CID:50.5 (15.8); Healthy controls:37.5 (13.4)	0
Haberman et al.-New York	2021	Multicenter, cohort	51 IMID and 26 controls	USA	White, Asian, Black, Hispanic	Female:36	Mean (range): IMID:56.15 (22, 79); Healthy controls: 49.2 (28, 74)	0
Haberman et al.-Erlangen	2021	NR	31 IMID and 182 controls	Germany	White, Asian, Black, Hispanic	IMID: Female:22; Healthy controls: 104	Mean (range): IMID:51.1 (24, 87); Healthy controls:40.8 (21, 65)	0
Mahil et al.	2021	Cohort	84 psoriasis and 17 controls	UK	White, Black, South Asian, Mixed	Female :45	Median (IQR):43 (31, 52)	0
Rabinowitz et al.	2022	Multicenter, prospective, observational	121 IBD and 89 controls	Israel	NA	Female: 60; Healthy controls: 62	38.8 (20.4)	NR
Rubbert et al.	2021	non-randomized, prospective, observational	51 RA and 20 Healthy controls	Switzerland	NA	Female:29	64.6 (11.5); Healthy control 41.7 (9.9)	0
Seyahi et al.	2021	Cross-sectional	104 IMID and 300 controls	Turkey	NA	Female:53	IMID: 42.2 (10.0); Healthy controls: 41.7 (9.90)	0
Signorelli et al.	2022	Prospective controlled phase 4	44 PAPS and 132 Healthy controls	Brazil	NA	Female: PAPS: 38; Healthy controls: 114	Median (IQR): PAPS: 46 (31, 73); Healthy controls 46 (31, 78)	4
Simon et al.	2021	Single center, observational, cohort	84 IMID and 182 controls	Germany	NA	Female: IMID: 55; Healthy controls: 14	53.1 (17.0); Healthy control:40.8 (12.0)	6
Simon et al.-no history	2022	Cohort	7 patients on RTX and 30 controls	Germany	NA	Female: 5; Healthy controls: 23	53.5(7.7); Healthy control:57.1(7.5)	0
Simon et al.-with history	2022	Cohort	6 patients on RTX and 30 controls	Germany	NA	Female: 5; Healthy controls: 13	62.5(12.8); Healthy control:61.0(16.6)	6; Healthy controls: 30
Valor-Mendez et al.	2021	Correspondence	10 AIDs and 10 controls	Germany	NA	Female:8	33(10)	0
Veestra et al.	2021	Correspondence	8 IMID and 66 controls	USA	NA	Female:7	NA	0
Vollenberg et al.-1st	2022	Multicenter, prospective, cohort	106 IBD and 42 Healthy controls	Germany	NA	Female: IBD:50	Median:46	0
Vollenberg et al.-2nd	2022	Multicenter, prospective, cohort	106 IBD and 42 Healthy controls	Germany	NA	Female: IBD:50	Median:46	0
Vollenberg et al.-3–6mon after 2nd	2022	Multicenter, prospective, cohort	106 IBD and 42 Healthy controls	Germany	NA	Female: IBD:50	Median:46	0
Wang et al.-SLE	2022	Multicenter, prospective, cohort	60 SLE and 35 Healthy controls	China	Asian	Female/male: SLE:2/60; Healthy controls: 3/32	SLE:40.08 (12.50); Healthy controls: 39.49 (10.23)	0
Wang et al.-RA	2022	Multicenter, prospective, cohort	70 RA and 35 Healthy controls	China	Asian	Female/male: RA: 6/70; Healthy controls: 3/35	RA :40.70(11.73); Healthy controls: 39.49 (10.23)	0
Wong et al.	2021	Single center, serosurvey	48 IBD and 14 Non-IBD controls, 29 PICR controls	USA	White, Non-White	NA	IBD:49.1(20.2); non-IBD control:35.2(9.4); PICR control:31.5 (10.3)	2
Achiron et al. (1)	2021	Prospective observational study	414 MS and 89 Healthy controls	USA	NR	Female: MS:282; Healthy controls:64	MS: 43.6 (9.9); Healthy controls:53.5 (15.4)	NR
Achiron et al. (3)	2022	Prospective, open-label, single-center, randomized clinical trial	20 MS	Israel	NR	Female:MS:12	49.55 (43.0, 58.1)	NR
Balcells et al.	2022	Multi-center, cohort	41 RD and 65 Healthy controls	Chile	NR	Female:30	Mean(range):51.7 (45.0)	NA
Barrios	2022	NR	17 CVID and 17 healthy controls	Germany	NR	Female: CVID:13; Healthy controls: 11	43.9 (17); Healthy controls:43.4 (16.5)	NR
Ben-Tov et al.	2021	Single center, retrospective cohort	12231 IBD and 3654 matched patients	Israel	NR	Female:6089, matched patients: 18120	47 (17)	0
Benucci et al. (1)	2022	NR	110 PsA and 96 Healthy controls	NR	NR	Female: PsA 71; Healthy controls: 65	61.72 (12) Healthy controls: 50.54 (11.66)	NR
Benucci et al.	2022	NR	200 RA and 96 Healthy controls	NR	NR	Female: RA 156; Healthy controls: 65	RA: 67.82 (13.29); Healthy controls:50.54 (11.66)	NR
Bergman et al.	2022	Single center, non-randomized prospective clinical trial	46 CVID and 85 Healthy controls	Sweden	NR	Female: CVID: 26; Healthy controls:28	CVID: 50.5 (2.2); Healthy controls:51.0 (1.9)	0
Bitoun et al.	2022	Observational cohort	62 AID	France	NR	Female:60	NA	NR
Bitzenhofer et al.	2022	Cross-sectional	39 humoral immunodeficiency and 19 Healthy controls	Switzerland	NR	Female: 28; Healthy controls:15	Median (IQR): 61.0 (52.5, 73.5); Healthy controls:53.0 (43.0, 63.0)	NR
Boekel et al. (3)	2022	Prespecified substudy of prospective multicenter cohort	4192 IMID and 822 Healthy controls	Netherlands	NR	Female: IMID: 2640; Healthy controls:549	IMID with immunosupressants:54 (13); IMID with immunosupressants:54 (14); Healthy controls:57 (14)	658
Bonfá et al.	2022	Prospective controlled study	910 ARD and 182 controls	Brazil	NR	NR	NR	NR
Brill et al.	2021	Single center, prospective cohort	72 MS and 50 Healthy controls	Israel	NR	Female:51; Healthy controls:25	MS on ocrelizumab: 47.9 (13.3); no treatment: 49 (13.4); Healthy controls: 45.3 (16)	NR
Brill et al. (1)	2022	Cohort	44 MS and 46 Healthy controls	Israel	NR	Female:33; Healthy controls:25	MS: 39.45 (12.88); Healthy controls: 45.3 (16)	0
Brill et al. (2)	2022	Cohort	67 MS and 30 Healthy controls	Israel	NR	Female:48; Healthy controls:16	MS:47.8 (13.5); Healthy controls:43.0 (14.8)	2
Brill et al.	2022	Single center, cohort	33 patients with MS and 40 Healthy controls	Israel	NR	Female:23; Healthy controls:24	MS:42.29 (11.28); Healthy controls:44.68 (14.94)	0
Bsteh et al.	2022	Multicenter, prospective observational cohort	456 MS and 116 Healthy controls	Austria	NR	Female:314; Healthy controls:82	MS:40.5(11.6); Healthy controls:41.4 (12.3)	0
Hadi et al.	2021	Multicenter, retrospective cohort	5562 IBD	USA	African American, Caucasian	Female: 3263	57.3 (17.5)	0
Khan et al.	2021	Multicenter, retrospective cohort	353 IBD	USA	White, Black, Hispanic, Other	Female:544; Male: 6777	Median (IQR): 71 (60, 75)	0
Classen et al.	2021	Single center retrospective cohort	72 IBD and 72 Healthy controls	Germany	NR	Female: 38; Healthy controls: 37	48.4 (15.2); Healthy controls: 46.3 (12.6)	3
Shehab et al.	2021	Multicenter, prospective cohort	58 IBD	Kuwait	NR	Female:25; Healthy controls: 27	IBD:40 (33, 52); Healthy controls:38 (30, 49)	NR
Caldera et al.	2021	Prospective cohort	122 IBD and 60 Healthy controls	USA	NR	Female:58; Healthy controls: 34	Median (IQR): IBD:33.2; Healthy controls:34	NR
Reuken et al.	2021	Single center, prospective cohort	28 IBD and 27 Healthy controls	Germany	NR	Female:13	Median (IQR):42(36, 59);	NR
Zhao et al.	2022	Multicenter, prospective cohort	42 RA and 26 Healthy controls	China	NR	Female:30; Healthy controls: 10	Median (IQR):56 (45.5, 60); Healthy controls:44.5 (36.26,53)	0
Zacharopoulou et al.	2022	Multicenter, prospective cohort	403 IBD and 124 Healthy controls	Greece	NR	Female:188; Healthy controls: 72	Median (IQR):45 (34, 56); Healthy controls:51 (48,54)	7
Yuki et al.	2022	Prospective controlled study	232 SLE and 58 Healthy controls	Brazil	White	Female: 208; Healthy controls: 52	Median (IQR):40.5 (18, 73); Healthy controls:41.5 (22,74)	NR
Lev-Tzion et al.	2022	Multiple national Insurance carriers, retrospective, and prospective cohort	4946 IBD and 4946 matched people	Israel	NA	Female: IBD: 2412; matched people: 2412	IBD:51 (16); matched people:51 (16)	NR
Medeiros-Ribeiro et al.	2022	Single center, prospective, controlled phase 4 study	251 ARD and 104 Healthy controls	Brazil	Caucasian race	Female: ARD: 235; Healthy controls: 94	Median (IQR): ARD:59 (50, 65); Healthy controls: 58 (49.8, 64)	0
Pasoto et al.	2022	Single-center, prospective controlled trial	51 Primary SjS and 102 Healthy controls	Brazil	White	Female: Primary SjS: 50; Healthy controls: 100	Primary SjS: 53.5 (11.7); Healthy controls:53.4 (11.4)	0
Sampaio-Barros et al.	2021	prospective study	51 SSc and 153 Healthy controls	Brazil	Caucasians, *n* = 30	Female: 3; Healthy controls: 9	Median (IQR): SSc:48 (38.5, 57); Healthy controls:48 (38, 57)	0
Fabris et al.	2022	Cohort	28 patients and 13 Healthy controls	Italy	NA	7	46 (9)	0
Tran et al.	2022	Multicenter, prospective cohort	181 IMID, 59 Healthy controls	Australia	African American or black, Arabic, Asian, Indian, Indigenous White	Female: 125; healthy controls:33;	Continue therapy:53.3 (14.4); Withhold therapy:55.4 (12.6); Healthy controls: 54.4 (12.6)	NR
Udaondo et al.	2021	Single center, longitudinal	40 adolescents with RD, 24 Healthy controls	Spain	NA	Female: RD: 22; Controls: 12	Mean: RD:14; controls:13;	8
Verstappen et al.	2022	Single center, longitudinal	67 patients with pSS and 33 Healthy controls	Netherlands	NA	Female: pSS:58; HC:33	pSS:59 (51, 67); HC:41 (33, 62)	NR
Firinu et al.	2022	Single center, prospective cohort	18 anti-CD20; 13 IMID; 18 Healthy Controls	Italy	NA	Female: Anti-CD20: 9; IMID: 8; Healthy Controls: 9	Anti-CD20:46 (17.2); IMID: 59 (26.3); Healthy Controls: 51.5 (24.2)	NR
Furer et al.	2022	Multicenter, prospective observational multicenter study	108 AIIRD-RTX patients; 122 immunocompetent controls	Israel	NA	Female: 83	Median (IQR): 65.5 (23, 88)	NR
Gallo et al.	2021	Multicenter, prospective, cohort	4 MS and 55 Healthy controls	Italy	NA	Female: 1	Mean: MS:40.25; Healthy Controls:41.2	NR
Jyssum et al.	2022	Multicenter, prospective, observational cohort	87 RA and 1114 Healthy controls	Norway	NA	Female: 854	60 (55, 67)	3
Kim et al.	2022	observational cohort	149 ARD and 94 Healthy controls	South Korea	NA	Female: ARD: 111; Healthy controls: 66	Median (IQR): ARD: 55.78 (37.0, 69.5); Healthy controls:38.5 (24, 64)	NR
Kornek et al.	2022	Single center, prospective cohort	82 patients and 82 Healthy controls	Austria	NA	Female: 23	Median (IQR): 40 (22)	4
Krbot Skoeic et al.	2022	NA	13 Patients and 11 Healthy controls	Croatia	NA	Female: 8; Healthy Controls:8	Patients:52.8 (9.7); Healthy Controls:50.7 (9.9)	NA
Mauro et al.	2022	Single center, observational cross-sectional cohort	237 RD and 232 Healthy controls	Italy	NA	Female: Patients:164; Healthy Controls:161	Mean (95%CI): IRD:57 (56, 59); Healthy controls:57 (56, 58)	13
Picchiant-Diamanti et al.	2021	Multi-center, parallel prospective cohort	35 RA and 167 Healthy controls	Italy	West Europe, East Europe, Africa, America	Female: RA: 27; Healthy controls:119	Median (IQR): RA: 59 (55, 65); Healthy controls: 42 (32,53)	NR
Shields et al.	2022	Cohort	36 RD and 36 Healthy controls	UK	NA	Female: 22; Healthy controls: NA	Median (IQR): RD: 65.0 (59.0, 70.75); Healthy controls: 66(61, 73)	1
Simader et al.	2021	observational cohort	99 RA and 46 SpA, 169 Healthy Controls	Austria	NA	Female: RA:36; SpA:22; Healthy controls:100	RA:56.15 (11.80); SpA/pSA:51.33 (12.92); Healthy controls:46.18 (12.56)	NR
Stefanski et al.	2022	Cohort	12 RA, 19 patients on RTX and 30 Healthy controls	Germany	NA	Female: Patients:23; Healthy Controls:15	Median (IQR): Patients: 62.0 (56.6, 79.5); Healthy controls: 57.0 (46.25,79.5)	NR
Medeiros-Ribeiro et al.	2021	prospective phase 4 controlled trial	910 ARD and 182 Healthy controls	Brazil	NA	210/700 42/140	Median (IQR): 51 (40, 60); Healthy controls: 50 (41, 60)	0
Ozdede et al.-CoronaVac	2022	Single center cross-sectional study	80 BS and 89 Healthy controls	Turkey	NA	Female: 42 Healthy controls: 36	BS: 43.9 (9.8); Healthy controls: NA	0
Ozdede et al.-BioNTech	2022	Single center cross-sectional study	86 BS and 76 Healthy controls	Turkey	NA	Female: 50/36 Healthy controls: 39	BS: 42.1 (9.4); Healthy controls: NA	0
Pellicano et al.	2022	Single center, prospective, cohort	90 SSc and 58 Healthy controls	Italy	NA	Female: 25; NA	50 (36, 61); NA	1
Sugihara et al.	2022	Multicenter prospective study	123 RMD and 43 Healthy controls	Japan	NA	Female: 24; Healthy controls: 14	65.6 (15.0); 50.4 (12.6)	0
Syversen et al.	2022	longitudinal observational study	1647 IMIDs and 1114 Healthy controls	Norway	NA	Female: 748; Healthy controls: 260	Median (IQR): 52 (40, 63); Healthy controls: 43 (32, 55)	0
Haidar et al.	2022	Multicenter, prospective observational cohort	1099 patients and 172 Healthy controls	USA	White; Non-White	Female: 74; Healthy controls: 43	Patients: 55.3 (14.8); Healthy controls: 44.2 (13.3)	0
Bergman et al.	2021	Single center, open-lable, non-randomized prospective clinical trial	90 PID and 30 Healthy controls	Sweden	NA	Female: 35; Healthy controls: 39	NA	0
Calderna et al.	2022	Multicenter, prospective, non-randomized study	158 IBD and 20 Healthy controls	USA	NA	Female: 79; Healthy controls: 9	Median (IQR): IBD: 42 (35,57); Healthy controls: 50 (42,58)	0
Capuano et al.	2022	observational prospective study	50 MS and 47 Healthy controls	Italy	NA	Female:27; Healthy controls: 19	NA; Healthy controls: 41.4 (13.2)	0
Chen et al.	2022	Cohort	389 ARID and 56 Healthy controls	China	NA	NA	NA	0
Delvino et al.	2021	Cohort	48 GCA and 140 Healthy controls	Italy	NA	Female:13; Healthy controls: NA	72.0 (5.2); Healthy controls: NA	0
Doherty et al.	2022	Multicenter prospective, observational cohort	270 IBD and 116 Healthy controls	Ireland	NA	Female:162; Healthy controls: 22	Median (IQR):40.6 (30.0, 49.4); Healthy controls: 34.9 (28.3, 49.6)	6
Duengelhoef et al.	2022	Prospective observational cohort	103 AIH, 64 PSC, and 61 PBC and 95 Healthy controls	Germany	NA	Female:63; Healthy controls: 23	NA	0
Ferri et al.	2022	Multicenter, prospective observational cohort	244 ASD and 502 Healthy controls	Italy	NA	Female:41; Healthy controls: 136	ASD: 62 (14); Healthy controls: 59 (14)	0
Firinu et al. (1)	2022	prospective observational study	102 IMID and 551 Healthy controls	Italy	NA	NA	NA	9
Giannoccaro et al.	2022	Multicenter, case-control, prospective study	39 MS and 273 Healthy controls	Italy	NA	Female:9; Healthy controls: 78	Median (IQR): 43 (31, 53); Healthy controls: 46 (38, 55)	NR
Giossi et al.	2022	Multiple national Insurance carriers, retrospective and prospective cohort	4946 IBD and 4946 matched people	Israel	NA	Female: IBD: 2412; matched people: 2412	IBD:51 (16); matched people:51 (16)	4
Shenoy et al.	2021	Cohort	102 AIRD and 34 non-AIRD	India	NA	Female: AIRD: 81; non-AIRD: 24	AIRD:52(12.33); non-AIRD:54.12(11.21)	0

*Note:* IQR: interquartile range; SD, Standard deviation; SARS-CoV-2, Severe Acute Respiratory Syndrome Coronavirus 2; IgG, immunoglobin G; IgG(S-RBD): IgG receptor binding domain (S-RBD); AZD1222: AstraZeneca-Oxford ChAdOx1 nCoV-19; NA: not available; NR: not reported; PsA, psoriatic arthritis; PAPS: primary antiphospholipid syndrome; MS: multiple sclerosis; CID, chronic inflammatory diseases; CVID, Common Variable Immunodeficiency; AIRD or ARD, autoimmune rheumatic disease; autoimmune rheumatic diseases; ASD, autoimmune systemic diseases; RD, rheumatic disease; RMD, Rheumatic musculoskeletal diseases; IMID, immune-mediated inflammatory disease; AID, autoinflammatory diseases; AIIRD, autoimmune inflammatory rheumatic diseases; RA, rheumatoid arthritis; SLE, systemic lupus erythematosus; PSC, primary sclerosing cholangitis; PBC, primary biliary cholangitis; RTX, rituximab; IBD, inflammatory bowel disease; SjS, Sjögren’s syndrome; SSc, systemic sclerosis; BS, Behçet’s syndrome.

**Table:** 

Study	Year	Treatment strategy	Disease	Vaccine subtype	Vaccine doses	Immunologic assay
Alexander et al.	2022	Thiopurine monotherapy, *n* = 64; IFX monotherapy, *n* = 49; IFX and thiopurine combination therapy, *n* = 56; Ustekinumab monotherapy, *n* = 49; Vedolizumab monotherapy, *n* = 50; Tofacitinib monotherapy, *n* = 19.	Crohn’s disease, *n* = 162; UC, *n* = 118; IBD-U, *n* = 7.	Pfizer-BioNTech, Moderna, Oxford-AstraZeneca	2	Roche Elecsys Anti-SARS-CoV-2 spike ECLIA
Boekel et al.-1st	2021	Prednisone monotherapy, MTX, TNFi, anti-CD20 therapy, monotherapy with prednisone	RA, *n* = 260; PsA, *n* = 68; Ankylosing spondylitis, *n* = 68; Axial or peripheral SpA, *n* = 6; JIA, *n* = 8; SLE, *n* = 33; Vasculitis, *n* = 11; PMR, *n* = 37; SjS, *n* = 33; Other RD, *n* = 103; MS, *n* = 58.	Pfizer, Moderna, AZ, J&J	1 & 2	ELISA
Boekel et al.-2nd	2021	Prednisone monotherapy, MTX, TNFi, anti-CD20 therapy, monotherapy with prednisone	RA, *n* = 260; PsA, *n* = 68; Ankylosing spondylitis, *n* = 68; Axial or peripheral SpA, *n* = 6; JIA, *n* = 8; SLE, *n* = 33; Vasculitis, *n* = 11; PMR, *n* = 37; SjS, *n* = 33; Other RD, *n* = 103; MS, *n* = 58.	Pfizer, Moderna, AZ, J&J	1 & 2	ELISA
Deepak et al.	2021	Prednisone monotherapy, *n* = 17; MTX, *n* = 29; HCQ, *n* = 30; AZA, *n* = 4; LEF, *n* = 2; Sulfasalazine, *n* = 7; Teriflunomide, *n* = 1; 6-MP, *n* = 2; JAKi (Tofacitinib, *n* = 10; Upadacitinib, *n* = 1); Biologic therapies(TNFi, *n* = 38; B-cell depleting therapies, *n* = 10; Belimumab, *n* = 3; Vedolizumab, *n* = 12); IL-12/23 or IL-23i (ABA, *n* = 2; TCZ, *n* = 1; Ibrutinib, *n* = 1); NSAIDs, *n* = 27; No immunosuppression, *n* = 9.	IBD, *n* = 42 (Crohn disease, *n* = 22; UC, *n* = 18; Other, *n* = 2); RA, *n* = 38; SpA, *n* = 20 (Axial SpA, *n* = 6; PsA/psoriasis, *n* = 10; IBD-associated arthritis, *n* = 4); Uveitis, *n* = 5; SLE, *n* = 15;Other CTD, *n* = 4; SjS, *n* = 8; Vasclitis, *n* = 5; Autoinflammatory syndrome, *n* = 2; MS, *n* = 9; Neuromyelitis optica, *n* = 1; IgG4-related disease, *n* = 2; Hidradenitis suppurativa, *n* = 1; Antiphospholipid syndrome, *n* = 1.	Pfizer & Moderna	2	ELISA and ELISpot assays
Edelman-Klapper et al.	2022	TNFi-α, *n* = 67; IFX, *n* = 34; ADA, *n* = 33; Vedolizumab, *n* = 26; Ustekinumab, *n* = 5; 5-ASA, *n* = 42; Steroids, *n* = 8; JAKi, *n* = 3; No treatment, *n* = 38.	Crohn’s disease, *n* = 122; UC, *n* = 53; ileal pouch anal-anastomosis, *n* = 6; IBD-U, *n* = 4.	Pfizer	1 & 2	Abbott architect i2000sr platform; ELISA
Ferri et al.	2021	GC, *n* = 204; MTX, *n* = 78; Sulfasalazine, *n* = 4; HCQ, *n* = 112; LEF, *n* = 4; MMF, *n* = 79; AZA, *n* = 22; TNFi, *n* = 23; ABA, *n* = 11; RTX, *n* = 26; IL6i, *n* = 21; JAKi, *n* = 14; Belimumab, *n* = 14.	ASD, *n* = 478.	BNT162b2 & mRNA-1273	2	Abbott Laboratories, IL
Furer et al.	2021	GC, *n* = 130; GC monotherapy, *n* = 13; MTX, *n* = 176; MTX monotherapy, *n* = 41; HCQ, *n* = 133, HCQ monotherapy, *n* = 50; LEF, *n* = 28; LEF monotherapy, *n* = 11; TNFi, *n* = 122; TNFi monotherapy, *n* = 121; TNFi + MTX, *n* = 29; IL6i, *n* = 37; IL6i monotherapy, *n* = 19; IL6i + MTX, *n* = 7; Anti-CD20, *n* = 87; Anti-CD20 monotherapy, *n* = 28; RTX+MTX, *n* = 14; IL-17i, *n* = 48; IL17i monotherapy, *n* = 37; IL17i + MTX, *n* = 7; ABA, *n* = 16; ABA monotherapy, *n* = 7; MMF, *n* = 28;ABA+MTX, *n* = 5; JAKi monotherapy, *n* = 21; JAKi + MTX, *n* = 24; Belimumab, *n* = 9.	RA, *n* = 263; PsA, *n* = 165; axial SpA, *n* = 68; SLE, *n* = 101; idiopathic inflammatory myositis, *n* = 19; antineutrophil cytoplasmic antibody, *n* = 26; large vessel vasculitis, *n* = 21.	Pfizer	2	LIAISON quantitative assay
Geisen et al.	2021	bDMARDs (Golimumab, *n* = 1; Certolizumab pegol, *n* = 3; Etanercept, *n* = 3; IFX, *n* = 3; TCZ, *n* = 1; Ixekizumab, *n* = 1; Vedolizumab, *n* = 1; ADA, *n* = 3; Secukinumab, *n* = 2; Ustekinumab, *n* = 1; Belimumab, *n* = 1); cDMARDs (HCQ, *n* = 3; LEF, *n* = 3, Sulfasalazine, *n* = 1; AZA, *n* = 1)	PsA, *n* = 2; RA, *n* = 7; Mixed CTD, *n* = 1; Spondyloarthropathy, *n* = 3; Sarcoidosis, *n* = 1; GCV, *n* = 1; Psoriasis, *n* = 4; Crohn’s disease, *n* = 4; SLE, *n* = 2; MS, =1; Myositis, *n* = 1.	Pfizer & Moderna	2	ELISA
Haberman et al.-New York	2021	MTX, *n* = 25; TNFi, *n* = 20; Other Anticytokines/JAKi, *n* = 10; other oral immunomodulators, *n* = 13.	PsA, *n* = 24; RA, *n* = 22; other, *n* = 5.	mRNA vaccine	2	ELISA
Haberman et al.-Erlangen	2021	MTX, *n* = 20; TNFi, *n* = 11;	IMID, *n* = 31.	BNT162b2	2	ELISA
Mahil et al.	2021	MTX monotherapy, *n* = 17; TNFi, *n* = 27; IL-17i, *n* = 15; IL-23i, *n* = 15.	Psoriasis, *n* = 84.	BNT162b2	1	ELISA
Rabinowitz et al.	2022	IFX, *n* = 29; ADA, *n* = 26; Vedolizumab, *n* = 23; Ustekinumab (5-ASA, *n* = 38; Steroids, *n* = 8); Immunomodulators, *n* = 12; JAKi, *n* = 5; No treatment, *n* = 29.	Crohn’s disease, *n* = 97; UC, *n* = 43; ileal pouch-anal anastomoisis, *n* = 7; IBD-U, *n* = 4.	BNT162b2	1 & 2	hematology analyzer
Rubbert et al.	2021	csDMARDs monotherapy, *n* = 16; bDMARDs, *n* = 25; bDMARDs monotherapy), *n* = 9; JAKi, *n* = 12; JAKi monotherapy, *n* = 5, Prednisone, *n* = 17; MTX, *n* = 28.	RA, *n* = 51.	Pfizer & Moderna	1 & 2	Roche Elecsys Anti-SARS CoV-2 spike subunit 1
Seyahi et al.	2021	Prednisolone, *n* = 17; Biological agents, *n* = 32; Conventional DMARDs, *n* = 27; Colchicine, *n* = 16; HCQ, *n* = 12.	RA, *n* = 11; CTD, *n* = 12; Vasculitis, *n* = 4; Spondyloarthropathy, *n* = 23; BS, *n* = 14; Familial Mediterranean fever, *n* = 9; IBD, *n* = 3; Other, *n* = 6.	CoronaVac	2	Roche Diagnostics International Ltd
Signorelli et al.	2022	VKA, *n* = 39; LMWH, *n* = 3; LDA, *n* = 8; HCQ, *n* = 17.	Antiphospholipid syndrome, *n* = 44.	Sinovac-CoronaVac	1 & 2	Indirect ELISA; SARS-CoV-2 sVNT Kit
Simon et al.	2021	No treatment, *n* = 24; GC, *n* = 10; csDMARDs monotherapy, *n* = 20; MTX, *n* = 16; HCQ, *n* = 3; Sulfasalazine, *n* = 1; bDMARDs/tsDMARDs, *n* = 36; TNFi, *n* = 11; IL6i, *n* = 3; IL-23i, *n* = 6; IL-17i, *n* = 7; JAKi, *n* = 6; Others, *n* = 3.	spondylarthritis, *n* = 27; RA, *n* = 25; IBD, *n* = 8; Psoriasis, *n* = 8; Systematic diseases, *n* = 16.	Pfizer	1 & 2	ELISA
Simon et al.-no history	2022	RTX, *n* = 8	spondylarthritis, *n* = 27; RA, *n* = 25; IBD, *n* = 8; Psoriasis, *n* = 8; Systematic diseases, *n* = 17.	Pfizer	2	ELISA, ELISpot IFN -γ enzyme-linked immunospot assay
Simon et al.-with history	2022	RTX, *n* = 6	spondylarthritis, *n* = 27; RA, *n* = 25; IBD, *n* = 8; Psoriasis, *n* = 8; Systematic diseases, *n* = 17.	Pfizer & Moderna	2	ELISA, (ELISpot) IFN -γ enzyme-linked immunospot assay
Valor-Mendez et al.	2021	IL-1 inhibitors, *n* = 10; Canakinumab, *n* = 8; Anakinra, *n* = 2.	adult-onset Still’s disease, *n* = 4; familial Mediterranean fever, *n* = 3; AID, *n* = 1.	Pfizer & Moderna	2	ELISA; cPASS
Veestra et al.	2021	bDMARD (Ixekizumab, *n* = 1); non-bDMARD (Tofacitinib, *n* = 1; AZA, *n* = 1; HCQ, *n* = 1; MTX, *n* = 1; MMF; corticosteroid; MTX and prednisone 5 mg; Prednisone, *n* = 1.	RA, *n* = 3; SLE, *n* = 4; hidradenitis suppurativa and leukocytoclastic vasculitis, *n* = 1; UC, *n* = 1; Psoriasis and PsA, *n* = 4.	Pfizer & Moderna	2	ELISA kit
Vollenberg et al.-1st	2022	Prednisolone, *n* = 11; Budesonide, *n* = 13; Mesalazine, *n* = 58; ADA, *n* = 21; IFX, *n* = 31; Vedolizumab, *n* = 15; Ustekinumab, *n* = 18; Others: *n* = 9.	Crohn’s disease, *n* = 119; UC, *n* = 70.	Pfizer & Moderna	1 &2	Abbott Diagnostics’; CMIA
Vollenberg et al.-2nd	2022	Prednisolone, *n* = 11; Budesonide, *n* = 13; Mesalazine, *n* = 58; ADA, *n* = 21; IFX, *n* = 31; Vedolizumab, *n* = 15; Ustekinumab, *n* = 18; Others: *n* = 9.	Crohn’s disease, *n* = 119; UC, *n* = 70.	Pfizer & Moderna	1 &2	Abbott Diagnostics’; CMIA
Vollenberg et al.-3–6mon after 2nd	2022	Prednisolone, *n* = 11; Budesonide, *n* = 13; Mesalazine, *n* = 58; ADA, *n* = 21; IFX, *n* = 31; Vedolizumab, *n* = 15; Ustekinumab, *n* = 18; Others: *n* = 9.	Crohn’s disease, *n* = 119; UC, *n* = 70.	Pfizer & Moderna	1 &2	Abbott Diagnostics’; CMIA
Wang et al.-SLE	2022	Prednisone, *n* = 55; HCQ, *n* = 56; MTX, *n* = 8; Ciclosporin, *n* = 11; LEF, *n* = 4; Alfacalcidol, *n* = 4; Rabeprazole, *n* = 3; Aspirin, *n* = 3.	SLE, *n* = 60.	Vero cell	2	CMIA
Wang et al.-RA	2022	Prednisone, *n* = 27; HCQ, *n* = 26; MTX, *n* = 40; LEF, *n* = 39; Alfacalcidol, *n* = 11; Iguratimod, *n* = 4; Total glucosides of paeon, *n* = 5	RA, *n* = 70.	Vero cell	2	CMIA
Wong et al.	2021	TNF antagonist monotherapy, *n* = 16; Vedolizumab monotherapy, *n* = 17; Vedolizumab combination therapy with thiopurine, *n* = 3; Ustekinumab, *n* = 4; Guselkumab, *n* = 1; steroids, *n* = 3; no medications, *n* = 5.	Crohn’s disease, *n* = 23; UC, *n* = 25.	Pfizer & Moderna	2	Siemens Healthineers COV2T and sCOVG assays, ELISA
Achiron et al. (1)	2021	Alemtuzumab, *n* = 22; Cladribine, *n* = 48; Dimethyl fumarate, *n* = 35; Fingolimod, *n* = 42; Natalizumab, *n* = 32; Ocrelizumab, *n* = 114; RTX, *n* = 6; Teriflunomide, *n* = 39.	MS, *n* = 414.	Pfizer	2	Anti-SARS-COV-2 ELISA IgG, ELISpotPLUS
Achiron et al. (3)	2022	Fingolimod, *n* = 20.	MS, *n* = 20.	Pfizer	2&3	DxI hematology analyzer, ISA IgG, Ficoll Hypaque
Balcells et al.	2022	Prednisone, *n* = 22; HCQ, *n* = 8; SSZ, *n* = 7; LEF, *n* = 10; MTX, *n* = 20; MMF, *n* = 1; TNFi, *n* = 40; Anti-IL6(TCZ), *n* = 1.	RA, *n* = 31; PsA, *n* = 9; JIA, *n* = 1.	CoronaVac vaccine	2	SARS-CoV-2 sVNT Kit, SARS-CoV-2 QuantiVac
Barrois	2022	NR	CVID, *n* = 17	Pfizer	2	ELISA
Ben-Tov et al.	2021	5-ASA or none, *n* = 8870; Corticosteroids, *n* = 447; TNFi, *n* = 1323; UST, *n* = 225; VDZ, *n* = 454; MTX, *n* = 170; Thiopurines, *n* = 591; Thiopurines and TNFi combination, *n* = 133.	UC, *n* = 6339; CD, *n* = 5442; Unspecified, *n* = 452.	BNT162b2	2	NR
Benucci et al. (1)	2022	Anti-IL17, *n* = 37; TNFi-α, *n* = 37; Etanercept, *n* = 26; MTX, *n* = 10.	PsA, *n* = 110	BNT162b2	1 & 2	IFN-γ ELISA
Benucci et al.	2022	b/tsDMARD (ABA, *n* = 35; ADA, *n* = 12; Bariticitinib, *n* = 15; Certolizumab, *n* = 6; Etanercept, *n* = 23; Golimumab, *n* = 1; IFX, *n* = 2; RTX, *n* = 18; Sarilumab, *n* = 9; TCZ, *n* = 52; Tofacitinib, *n* = 4; Upadacitinib, *n* = 7; JAKi, *n* = 24; TNFi, *n* = 43); MTX, *n* = 78.	RA, *n* = 200	BNT162b2	2	IFN-γ ELISA
Bergman et al.	2022	Immunoglobulin replacement therapy, *n* = 44; Steroids, *n* = 4; RTX, *n* = 2.	CVID, *n* = 46.	Pfzer/BioNTech	2	IFN-γ ELISA
Bitoun et al.	2022	Corticosteroids, *n* = 23; MTX, *n* = 36.	RA, *n* = 42; Other AID, *n* = 20.	NR	2	ELISA
Bitzenhofer et al.	2022	Ibrutinib/RTX, *n* = 2; RTX, *n* = 2; Certolizumab, *n* = 1; Etanercept, *n* = 1; Ocrelizumab, *n* = 1; MTX/prednisolone, *n* = 1; MMF, *n* = 1; intravenous immunoglobulin substitution, *n* = 36; subcutaneous immunoglobulin substitution, *n* = 3.	CVID, *n* = 26; IgG deficiency, *n* = 2; Secondary immunodeficiency (drugs, neoplastic disease), *n* = 2; IgG subclass deficiency, *n* = 9.	Pfizer/BionTech or Spikevax, Moderna	2&3	Abbott SARS-CoV-2 nucleoprotein assay and SARS-CoV-2 S1/S2 IgG assay
Boekel et al. (3)	2022	MTX, *n* = 992; TNFi, *n* = 929; Anti-CD20, *n* = 266; MMF, *n* = 105; S1PMs, *n* = 66; Other IS, *n* = 432.	RD, *n* = 2531; Neurological, *n* = 675; Gastroenterological, *n* = 605; Dermatological, *n* = 381.	Oxford-AstraZeneca, Pfizer-BioNTech, mRNA-1273, Moderna, J & J	2&3	ELISA
Bonfá et al.	2022	NR	NR	CoronaVac	2	NR
Brill et al.	2021	Ocrelizumab, *n* = 49; No treatment, *n* = 23.	MS, *n* = 49.	Pfizer/BioNTech	2	Liaison SARS-CoV-2 S1/S2 IgG, Architect SARS-CoV-2 IgG II Quant assay, T-SPOT Discovery SARS-CoV-2
Brill et al. (1)	2022	Cladribine tablets, *n* = 36.	MS, *n* = 46.	BTN162b2, Pfizer/BioNTech	2 or 3	Architect SARS-CoV-2 IgG II Quant assay
Brill et al. (2)	2022	Cladribine tablets, *n* = 34.	MS, *n* = 67.	BTN162b2, Pfizer/BioNTech	2	SARS-CoV-2 S1/S2 IgG assay; SARS-CoV-2 RBD IgG Architect assay
Brill et al.	2022	Ocrelizumab, *n* = 33.	MS, *n* = 33.	BTN162b2, Pfizer/BioNTech	2&3	spike RBD Architect SARS-CoV-2 IgG II Quant assay, T-SPOT Discovery SARS-CoV-2
Bsteh et al.	2022	No treatment, *n* = 91; immunomodulating DMT, *n* = 139 (IM-DMT: Dimethyl fumarate, *n* = 63; Glatiramer acetate, *n* = 20; IFN-β preparations, *n* = 22; Natalizumab, *n* = 24; Teriflunomide, *n* = 10); Immunosuppressive DMT (Alemtuzumab, *n* = 8; Cladribine, *n* = 116; Fingolimod, *n* = 25; Ocrelizumab, Ozanimod, RTX or Siponimod); S1PMs, *n* = 226.	MS, *n* = 456.	mRNA vaccine, Vector-based vaccine	1	Anti-SARS-CoV-2-QuantiVac ELISA
Hadi et al.	2021	biologics/thiopurines, *n* = 2939.	Crohn’s disease, *n* = 2629; UC, *n* = 2933.	BNT162b2 & mRNA-1273	1 & 2	Roche Elecsys Anti-SARS-CoV-2 spike immunoassay, ELISA
Khan et al.	2021	5-ASA alone, *n* = 4022; Thiopurine, *n* = 793; TNFi alone, *n* = 1374; TNFi + IM, *n* = 307; Vedolizumab, *n* = 529; Ustekinumab, *n* = 75; Tofacitinib, *n* = 49; MTX, *n* = 172.	Crohn’s disease, *n* = 2746; UC, *n* = 4575.	BNT162b2, mRNA-1273, Ad26.CoV2.S	1 & 2	NR
Classen et al.	2021	Immunosuppression total, *n* = 65; Steroids, *n* = 2; Mesalazine, *n* = 24; AZA, *n* = 1; MTX, *n* = 1; TNF blocker, *n* = 27; IL, *n* = 19; Ustekinumab, *n* = 14.	Crohn’s disease, *n* = 40; UC, *n* = 32.	BNT162b2, mRNA-1273, Ad26.CoV2.S	1 & 2&3	Elecsys Anti-SARS-CoV-2 spike
Shehab et al.	2021	NR	Crohn’s disease, *n* = 34; UC, *n* = 24.	BNT162b2	2	ELISA
Caldera et al.	2021	No therapy, *n* = 8; Mesalazine, *n* = 10; Vedolizumab monotherapy, *n* = 10; Thiopurine, *n* = 6; TNFi therapy, *n* = 46; TNFi combination, *n* = 19; Tofacitinib, *n* = 6; Ustekinumab, *n* = 11; Corticosteroid therapy, *n* = 5.	Crohn’s disease, *n* = 85; UC, *n* = 37.	BNT162b2, mRNA-1273	2	LabCorp’s Cov2Quant IgG assay
Reuken et al.	2021	Any immunosuppression, *n* = 28; Steroid, *n* = 2; TNF-antibodies, *n* = 9; Vedolizumab, *n* = 3; Ustekinumb, *n* = 8; AZA, *n* = 3; MMF, *n* = 2; Tofacitinib, *n* = 1; Tacrolimus, *n* = 3.	Crohn’s disease, *n* = 17; UC, *n* = 10.	BNT162b2, AstraZeneca	2	Liaison SARS-CoV-2 Trimerics IgG CLIA
Zhao et al.	2022	csDMARDs, *n* = 31; bDMARSs, *n* = 17; JAKi, *n* = 9; Prednisone, *n* = 11; Tripterygium glycosides tablet, *n* = 6.	RA, *n* = 42.	inactivated SARS-CoV-2 vaccines	2	ELISA
Zacharopoulou et al.	2022	5-ASA, *n* = 135; Systematic corticosteroids, *n* = 15; Thiopurine, *n* = 53; MTX, *n* = 24; IFX, *n* = 134; ADA, *n* = 51; Golimumab, *n* = 3; Vedolizumab, *n* = 71; Ustekinumb, *n* = 33; Tofacitinib, *n* = 38; Immunomodulator, *n* = 28; TNFi-α, *n* = 153; Biologic monotherapy, *n* = 247; Two immunosuppressive agents, *n* = 55; Three immunosuppressive agents, *n* = 2; No immunosuppression, *n* = 70.	Crohn’s disease, *n* = 237; UC, *n* = 153; IBD-U, *n* = 4; Ileal pouch, *n* = 7.	Pfizer-BioNTech, Moderna, AstraZeneca, J&J	2	ELISA
Yuki et al.	2022	HCQ, *n* = 187; Prednisone, *n* = 126; Immunosuppressive drugs, *n* = 173; MMF, *n* = 73; AZA, *n* = 62; MTX, *n* = 25; CNI, *n* = 11; Cyclophosphamide, *n* = 6; LEF, *n* = 3; Belimumab, *n* = 32.	SLE, *n* = 232.	Sinovac-CoronaVac	2	ELISA, SARS-CoV-2S1/S2 IgG
Lev-Tzion et al.	2022	Mesalamine, *n* = 1441, Corticosteroid, *n* = 203; Immunomodulator, *n* = 294; TNFi, *n* = 487; Vedolizumab, *n* = 185; Ustekinumab, *n* = 96; Tofacitinib, *n* = 28.	Crohn’s disease, *n* = 2447; UC, *n* = 2499.	Pfizer-BioNTech	2	NR
Medeiros-Ribeiro et al.	2022	Prednisone, *n* = 157; DMARD, *n* = 2; No current DMARD, *n* = 7; DMARD monotherapy, *n* = 66; MTX, *n* = 117; LEF, *n* = 91; HCQ, *n* = 35; Sulfasalazine, *n* = 30; Tofacitinib, *n* = 19; TNFi, *n* = 58; ABA, *n* = 54; TCZ, *n* = 47.	RA, *n* = 260	Sinovac-CoronaVac	2	Indirect ELISA; SARS-CoV-2 sVNT Kit
Pasoto et al.	2022	HCQ, *n* = 31; prednisone, *n* = 14; AZA, *n* = 11; MMF, *n* = 7; MTX, *n* = 4; LEF, *n* = 1; ABA, *n* = 1, Ustekinumab, *n* = 1.	Primary SjS, *n* = 51.	Sinovac-CoronaVac	1 & 2	Indirect ELISA, SARS-CoV-2 sVNT Kit
Sampaio-Barros et al.	2021	HCQ, *n* = 3; Prednisone, *n* = 6; Immunosuppressive, *n* = 37; Monotherapy, *n* = 33; MMF, *n* = 27; MTX, *n* = 5; AZA, *n* = 4; LEF, *n* = 2; RTX, *n* = 1.	Diffuse SSc, *n* = 35; Limited SSc, *n* = 16	Sinovac-CoronaVac	2	Indirect ELISA, cPass sVNT Kit, GenScript
Fabris et al.	2022	RTX, *n* = 11; Belimumab, *n* = 17.	ANCA-associated vasculitis, *n* = 6; Mixed CTD, *n* = 2; Anti-synthetase syndrome, *n* = 2; Dermatomyositis, *n* = 1; SLE, *n* = 17.	mRNA-1273, BNT162b2	2	iFlash-SARS-CoV-2; Elecsys anti-SARS-CoV-2 ECLIA; IFN-γ
Tran et al.	2022	csDMARD, *n* = 58; bDMARD, *n* = 63; tsDMARD, *n* = 60	IMID, *n* = 181	CHadOx1nCov-19 (AstraZeneca); Pfizer	2	Siemens ADVIA Centaur sCOVG assay
Udaondo et al.	2021	ADA, *n* = 11; Etanercept, *n* = 9; IFX, *n* = 3; MMF, *n* = 5; Baricitinib, *n* = 5; TCZ, *n* = 1; CsA, *n* = 1; MTX, *n* = 14.	Crohn’s disease, BS	BNT162b2	2	CLIA
Verstappen et al.	2022	NA	primary SjS, *n* = 67.	BNT162b2, Astrazeneca, Moderna, J&J	2	ELIspotassay, MIA
Firinu et al.	2022	Anti-CD20, *n* = 18; csDMARDs, *n* = 10; Combination of MTX plus anti TNF, *n* = 3.	IMID, *n* = 13.	Pfizer/BioNTech BNT162b2	2&3	LIAISON SARS-CoV-2 TrimericS IgG assay, ELISA
Furer et al.	2022	csDMARDs, *n* = 34; MTX, *n* = 16; Prednisone, *n* = 54; LEF, *n* = 2; MMF, *n* = 5; IVIG, *n* = 9.	RA, *n* = 49; SLE, *n* = 11; ANCA-associated vasculitis, *n* = 23; Other systemic vasculitis, *n* = 6; idiopathic inflammatory myopathy, *n* = 18.	BNT162b2	2	SARS-CoV-2 IgG II Quant assay, LIAISON
Gallo et al.	2021	Cladribine, *n* = 1; IFN-β, *n* = 3; Teriflunomide, *n* = 1; Fingolimod, *n* = 3; Natalizumab, *n* = 2; Dimethyl Fumarate, *n* = 1.	MS, *n* = 4	BNT162b2	2	LIAISON SARS-CoV-2 TrimericS-IgG assay
Jyssum et al.	2022	RTX, *n* = 47; Prednisolone, *n* = 19; MTX, *n* = 64; LEF *n* = 9; Sulfasalazine, *n* = 4.	RA, *n* = 87	Pfizer/BioNtech, Moderna, AstraZeneca	2&3	Aminefunctionalized polymer beads
Kim et al.	2022	Steroid *n* = 62; HCQ, *n* = 42; MTX, *n* = 58; LEF, *n* = 29; Sulfasalazine, *n* = 2; MMF, *n* = 17; CNI, *n* = 23; AZA, *n* = 23; Cyclophosphamide, *n* = 2; JAKi, *n* = 3; TNFi, *n* = 17; TCZ, *n* = 3; Belimumab, *n* = 1.	SLE, *n* = 43; RA, *n* = 62; AS, *n* = 11; BS, *n* = 10; AOSD, *n* = 6.	BNT162b2, mRNA1273, AZD1222, Ad26.COV2.S	2&3	Euroimmun anti-SARSCoV-2 ELISA; GenScript, cPass surrogate virus neutralisation test; Euroimmun IGRA
Kornek et al.	2022	RTX, *n* = 76; Ocrelizumab, *n* = 6; AZA, *n* = 3; TCZ, *n* = 3, Oral prednisone, *n* = 2; Subcutaneous or intravenous immunoglobulins, *n* = 2; Eculizumab, *n* = 1; MMF, *n* = 1.	MS, *n* = 64; Neuromyelitis optica spectrum disorders, *n* = 7; Myasthenic syndromes, *n* = 7; Autoimmune encephalitis, *n* = 2; Chronic inflammatory demyelinating polyneuropathy, *n* = 2	Pfizer/BioNTech, Moderna	NR	Cobas e801 analyzers, IFN-γ ELISpot
Krbot Skoeic et al.	2022	Siponimod, *n* = 13.	secondary progressive MS	Pfizer, Moderna, AZD1222	2	Elecsys Anti-SARSCoV-2 S assay
Mauro et al.	2022	csDMARD, *n* = 154; MTX, *n* = 94; HCQ, *n* = 14; MMF, *n* = 16; AZA, *n* = 16; SSZ, *n* = 9; LEF, *n* = 2; Colchicine, *n* = 3; bDMARD and tsDMARD, *n* = 118; TNFi, *n* = 49; ABA, *n* = 15; UST, *n* = 7; IL-17i, *n* = 14; IL-6i, *n* = 12; IL-1i, *n* = 2; RTX, *n* = 4; BEL, *n* = 1; PDE4i, *n* = 1- JAKi, *n* = 18.	AID, *n* = 4; CTD, *n* = 62; RA, *n* = 86; Spondylarthritis, *n* = 71; Vasculitis, *n* = 14.	BNT162b2	2	Liaison SARS-CoV-2 TrimericS IgG CLIA
Picchiant-Diamanti et al.	2021	TNF-α-inhibitors +/- DMARD, *n* = 7; IL-6i+/-DMARD/CCS, *n* = 8; CTLA-4-inhibitors +/-DMARD/CCS, *n* = 13; DMARD +/- CCS, *n* = 7.	RA, *n* = 35.	BNT162b2	2	SARS − CoV − 2 PepTivato
Shields et al.	2022	B-cell-depleting agents (i.e. RTX or Obinutuzumab)	Rheumatology, *n* = 36.	AZD1222, Pfizer/BioNTech	NA	IgG/A/M anti-SARS-CoV-2 ELISA
Simader et al.	2021	MTX, *n* = 54; LEF, *n* = 5; AZA, *n* = 2; HCQ, *n* = 4; Salazopyrin, *n* = 4; ADA, *n* = 17; Certolizumab, *n* = 2; Etanercept, *n* = 5; Golumumab, *n* = 11; IFX, *n* = 4; Secukinumab, *n* = 5; Ixekizumab, *n* = 4; TCZ, *n* = 3; Baricitinib, *n* = 3; Upadacitinib, *n* = 3; Filgotinib, *n* = 1.	RA, *n* = 53; SpA, *n* = 46	mRNA vaccine	2	The Elecsys Anti-SARS-CoV-2 S immunoassay
Stefanski et al.	2022	MTX, *n* = 12; LEF, *n* = 1; Sulfasalazine, *n* = 1; AZA, *n* = 1; JAKi, *n* = 6; TNFi, *n* = 1; ABA, *n* = 3; Prednisolone, *n* = 11.	RA, *n* = 12; Patients on RTX, *n* = 19	BNT162b2, mRNA-1273, ChAdOx1	1 & 2	ELISA
Medeiros-Ribeiro et al.	2021	Prednisone, *n* = 348; HCQ, *n* = 269; Sulfasalazine, *n* = 73; MTX, *n* = 229; LEF, *n* = 130; MMF, *n* = 119; AZA, *n* = 109; Tofacitinib, *n* = 19; Cyclophosphamide, *n* = 10; Tacrolimus, *n* = 10;CsA, *n* = 9; Biologic therapy, *n* = 321; TNFi, *n* = 138; ABA, *n* = 51; TCZ, *n* = 50; Belimumab, *n* = 30; Secukinumab, *n* = 29; RTX, *n* = 19; Ustekinumab, *n* = 5.	Chronic inflammatory arthritis (RA, axSpA, PsA), *n* = 451; Other ARD (SLE, primary vasculitis, SSc, pSSj, IIM, PAPS), *n* = 459.	inactivated vaccine	2	ELISA, SARS-CoV-2 sVNT Kit
Ozdede et al.-CoronaVac	2022	Colchicine, *n* = 39; Prednisolone, *n* = 14; non-bDMARDs, *n* = 49; AZA, *n* = 44; Other DMARDs, *n* = 7; Anti-TNF agents, *n* = 33.	NA	Sinovac-CoronaVac	2	Elecsys Anti-SARS-CoV-2 S assay
Ozdede et al.-BioNTech	2022	Colchicine, *n* = 44; Prednisolone, *n* = 17; non-bDMARDs, *n* = 50; AZA, *n* = 42; Other bDMARDs, *n* = 9; Anti-TNF agents, *n* = 33.	NA	Pfizer/BioNTech	2	Elecsys Anti-SARS-CoV-2 S assay
Pellicano et al.	2022	Immunosuppressive therapies, *n* = 31; MTX, *n* = 6; MMF, *n* = 4; RTX, *n* = 8.	NA	Pfizer-BioNTech	2	LIAISON SARS-CoV-2 TrimericS IgG assay
Sugihara et al.	2022	Conv IS, *n* = 67; MTX, *n* = 43; AZA, *n* = 9; MMF, *n* = 2; Mizoribine, *n* = 2; Tacrolimus, *n* = 16;CsA, *n* = 7; TNFi, *n* = 10; IL-6i, *n* = 5; RTX, *n* = 6; ABA, *n* = 7; IL-17i or IL-23i, *n* = 6; Mepolizumab, *n* = 1; JAKi, *n* = 6; Baricitinib, *n* = 6; Glucocorticoids, *n* = 62.	RA, *n* = 54; SLE, *n* = 8; Antiphospholipid syndrome, *n* = 5; SjS, *n* = 24; Systemic sclerosis, *n* = 4; Polymyositis/dermatomyositis, *n* = 9; Anti-neutrophil cytoplasmic antibody, *n* = 10; IgG4-related disease, *n* = 11; Spondylarthritis, *n* = 11; Polymyalgia rheumatica *n* = 1, BS, *n* = 2, Takayasu’s arteritis *n* = 2, IgA vasculitis *n* = 1, relapsing polychondritis *n* = 1, undifferentiated CTD, *n* = 1, fibromyalgia syndrome *n* = 2, adult-onset Still’s disease *n* = 1, TAFRO syndrome *n* = 1; macrophage activating syndrome *n* = 1, hypergammaglobulinemia, *n* = 1.	BNT162b2	2	Elecsys Anti-SARS-CoV-2 S RUO
Syversen et al.	2022	MTX, *n* = 348; Vedolizumab, *n* = 55; JAKi, *n* = 50; Ustekinumab, *n* = 34; TCZ, *n* = 32; ABA, *n* = 15; Secukinumab, *n* = 13.	RA, *n* = 566; PsA, *n* = 295; Spondylarthritis, *n* = 305; Ulcerative colitis, *n* = 195; Crohn’s disease, *n* = 280.	ChAdOx1, BNT162b2, mRNA-1273	2 or 3	SARS–CoV-2 microneutralization assay
Haidar et al.	2022	TNFα-i, *n* = 111; Antimetabolites, *n* = 61; CNI, *n* = 4; Mercaptopurine, *n* = 9; JAKi, *n* = 13; HCQ, *n* = 42; MTX, *n* = 55; ILi, *n* = 9; Anti-CD20 therapy, *n* = 10.	MS, *n* = 6; IBD, *n* = 100; Hashimoto’s, *n* = 13; Interstitial lung disease, *n* = 7; RA, *n* = 55; Myositis, *n* = 6; SjS, *n* = 25; Scleroderma, *n* = 10; Lupus, *n* = 17; Vasculitis, *n* = 8; PsA, *n* = 20; Ankylosing spondylitis, *n* = 10.	Pfizer, Moderna, J&J, Ad26.COV2.S	2	Beckman Coulter SARS-CoV-2 platform
Bergman et al.	2021	Corticosteroids, *n* = 12; Other immunosuppressive agents, *n* = 13.	CVID, *n* = 50; XLA, *n* = 4; Low number or defect T-cell function, *n* = 14; Monogenic diseases, *n* = 10; Other with expected normal response, *n* = 12.	Pfizer/BioNTech	2	quantitative test Elecsys AntiSARS-CoV-2 S
Calderna et al.	2022	Mesalamine, *n* = 18; Vedolizumab, *n* = 25; Thiopurine, *n* = 9; Anti-TNF monotherapy, *n* = 61; Anti-TNF combination, *n* = 13; Ustekinumab, *n* = 16; Tofacitinib, *n* = 6; Corticosteroid therapy, *n* = 10.	Crohn’s Disease, *n* = 106; Ulcerative colitis, *n* = 52	Moderna, Pfizer	2	LabCorp’s Cov2Quant IgG assay; Roche anti nucleocapsid; The Elecsys Anti-SARS-CoV-2 S ECLIA
Capuano et al.	2022	Ocrelizumab, *n* = 32; Fingolimod, *n* = 27.	NA	BNT162b2	1	SARS-CoV-2 TrimericS-IgG assay
Chen et al.	2022	NA	NA	Oxford-AstraZeneca Moderna	2	Elecsys AntiSARS-CoV-2 S assay
Delvino et al.	2021	glucocorticoids, *n* = 44; MTX, *n* = 17; TCZ, *n* = 5.	Cranial-GCA, *n* = 29; Large-vessel-GCA, *n* = 9; Cranial and large-vessel GCA, *n* = 10	BNT162b2	2	LIAISON SARS-CoV-2 S1/S2 IgG
Doherty et al.	2022	5-ASA, *n* = 31; Anti-TNF therapy, *n* = 145; Anti-integrins, *n* = 14; Anti-IL 12/23, *n* = 8; Immunomodulators, *n* = 16; Steroids, *n* = 9; JAKi, *n* = 4.	NA	Moderna, BNT162b2, AstraZeneca Ad26.CoV2.S	2	SARS-CoV-2 IgG 75 CMIA
Duengelhoef et al.	2022	Steroids, *n* = 42; AZA, *n* = 65; MMF, *n* = 7.	AIH, *n* = 103; PSC, *n* = 64; PBC, *n* = 61	BNT162b2, Pfizer/BioNTech Moderna	2	DiaSorin LIAISON and Roche immunoassays
Ferri et al.	2022	Immunomodulatory treatment	RA, *n* = 48; SLE, *n* = 16; SSc, *n* = 153; CV, *n* = 21; Other Vasculitis, *n* = 4.	BNT162b2, mRNA-1273	2	SARS-CoV-2 IgG II Quant antibody test
Firinu et al. (1)	2022	Steroids, *n* = 31; IVIG or plasmapheresis, *n* = 25; AZA, *n* = 31; Ocrelizumab, *n* = 21; RTX, *n* = 25; Cladribine, *n* = 3; Natalizumab, *n* = 24; Fingolimod, *n* = 9; Glatiramer, *n* = 7; Interferon, *n* = 2; Teriflunomide, *n* = 5.	SLE, *n* = 22; RA, *n* = 22; Psoriasis, *n* = 21; Miscellaneous systemic disorders, *n* = 19; IBD, *n* = 6; MS, *n* = 7.	BNT162b2	2	Snibe Diagnostics, PRNT
Giannoccaro et al.	2022	Dimethyl fumarate, *n* = 15; Fingolimod, *n* = 5; Teriflunomide, *n* = 5; Interferons, *n* = 4; Glatiramer acetate, *n* = 3; Ocrelizumab, *n* = 3; Cladribine, *n* = 2; Alemtuzumab, *n* = 1; Natalizumab, *n* = 1.	MG, *n* = 88; MS, *n* = 169; CIDP, *n* = 34; Other, *n* = 9.	BNT162b2, mRNA-1273	2	Elecsys anti-SARS-CoV-2 ECLIA assay
Giossi et al.	2022	Mesalamine, *n* = 1441, Corticosteroid, *n* = 203; Immunomodulator, *n* = 294; Anti-TNF, *n* = 487; Vedolizumab, *n* = 185; Ustekinumab, *n* = 96; Tofacitinib, *n* = 28.	NA	BNT162b2	2	SARS-CoV-2 IgG II Quant
Shenoy et al.	2021	Methotrexate, *n* = 58; Sulfasalazine, *n* = 20; Leflunomide, *n* = 9; Hydroxychloroquine, *n* = 71; Tofacitinib, *n* = 6; Mycophenolate, *n* = 5; Tacrolimus, *n* = 2; Azathioprine, *n* = 2; Iguratiimod, *n* = 3; Apremilast, *n* = 3; Rituximab, *n* = 6; Adalimumab, *n* = 1; Oral steroids, *n* = 27.	Rheumatoid arthritis, *n* = 38; Palindromic Rheumatism, *n* = 17; Inflammatory Polyarthritis, *n* = 16; Spondyloarthropathy, *n* = 13; SLE, *n* = 9; Vasculitis, *n* = 5; Scleroderma, *n* = 3; Myosistis, *n* = 1.	BBV152	2	CLIA

*Note:* SARS-CoV-2, Severe Acute Respiratory Syndrome Coronavirus 2; IgG, immunoglobin G; IgG(S-RBD): IgG receptor binding domain (S-RBD); AZD1222: AstraZeneca-Oxford ChAdOx1 nCoV-19; J&J, Johnson & Johnson; NSAIDs, Non-Steroidal Anti-inflammatory Drugs; DMT, disease-modifying treatment; IMTs, Immune-modifying therapies; MMF, mycophenolate mofetil; IFX, infliximab; 5-ASA, 5-aminosalicylic acid; ADA: adalimumab; GC: glucocorticoids; ABA, abatacept; anti-CD20, CD-20 inhibitors; IL, interleukin IL6i, interleukin 6 inhibitors; IL17i, interleukin 17 inhibitors; IL23i, interleukin 23 inhibitors; JAKi, Janus kinase inhibitors; MTX, methotrexate; TNFi, tumour necrosis factor inhibitors; SSZ, salazopyrine; HCQ, hydroxychloroquine; LEF, leflunomide; CsA, cyclosporine; b/ts, biologic/targeted synthetic; bDMARD, biological disease-modifying anti-rheumatic drug; csDMARD, conventional synthetic disease-modifying anti-rheumatic drug; CTLA4-Ig, cytotoxic T-lymphocyte-associated protein-4 immunoglobulin; VKA, vitamin K antagonist, LMWH, low-molecular-weight heparin; LDA, low dose aspirin; AZA, azathioprine; 6MP, 6-mercaptopurine; MP, mercaptopurine; RTX, rituximab; S1PMs, spingosin 1 receptor modulators; SSZ, sulphasalazine; IFN-β, Interferon β; PDE4i, Phosphodiesterase-4 inhibitor; CNI, calcineurin inhibitor; TCZ, tocilizumab; PsA, psoriatic arthritis; PAPS: primary antiphospholipid syndrome; PSC, primary sclerosing cholangitis; PBC, primary biliary cholangitis; MS: multiple sclerosis; CID, chronic inflammatory diseases; CVID, Common Variable Immunodeficiency; CTD, connective tissue disease; AIH, autoimmune hepatitis; AID, autoinflammatory diseases; ARD, autoimmune rheumatic diseases; ASD, autoimmune systemic diseases; RA, Rheumatoid arthritis; RD, rheumatic disease; RMD, Rheumatic musculoskeletal diseases; IMID, immune-mediated inflammatory disease; AIIRD, autoimmune inflammatory rheumatic diseases; SLE, systemic lupus erythematosus; SjS, Sjögren’s syndrome; PID, primary immunodeficiency; IBD, inflammatory bowel disease; IBD-U, inflammatory bowel disease-unclassified, UC, ulcerative colitis; GCA, giant cell arteritis; GCV, giant cell vasculitis; PMR, polymyalgia rheumatica; SpA, spondyloarthritis; JIA, Juvenile Idiopathic Arthritis; JDM, Juvenile Dermatomyositis; SSc, systemic sclerosis; ANCA, antineutrophil cytoplasmic antibody, MG, Myasthenia gravis; CIDP, chronic inflammatory neuropathy; XLA, X-linked agammaglobulinemia; IS, immunosuppressant; IVIG, intravenous immunoglobulin; EIA, enzyme immunoassay; CMIA, chemiluminescent immunoassay microparticles; sVNT, Surrogate Virus Neutralization Test; CLIA, chemiluminescent immunoassay; ELISA, Enzyme-linked immunosorbent assay; ECLIA, electrochemiluminescence immunoassay analyzer; t; IGRA, Interferon Gamma Release Assay; PRNT, plaque reduction neutralization test; NA: not available; NR: not reported.

### Quality assessment

The quality of the non-randomized studies was assessed using the NIH quality assessment tool. A total of 17 studies were assessed as good quality with a score of ≥11, whereas as the rest of studies scored <11 score, indicating fair quality (Table 2).

**Table 2.** Quality assessment of the studies using NIH tool.

Study	Year	Item 1	Item 2	Item 3	Item 4	Item 5	Item 6	Item 7	Item 8	Item 9	Item 10	Item 11	Item 12	Item 13	Item 14	Score	Grade
Alexander et al.	2022	1	1	1	0	1	1	1	0	1	0	1	0	NA	0	8	Fair
Boekel et al.-1st	2021	1	1	1	0	1	1	1	1	0	1	1	0	NA	1	10	Fair
Deepak et al.	2021	1	1	1	0	1	0	1	0	1	0	1	1	NA	0	8	Fair
Edelman-Klapper et al.	2022	1	1	1	1	1	0	1	1	1	1	1	0	0	NA	10	Fair
Ferri et al.	2021	1	1	1	1	1	NA	1	0	1	0	1	0	0	0	8	Fair
Furer et al.	2021	1	1	1	1	1	0	1	1	1	0	1	NA	NA	1	10	Fair
Geisen et al.	2021	1	0	1	0	0	1	0	1	1	1	1	0	NA	0	7	Fair
Haberman et al.	2021	1	1	1	NA	1	1	0	0	1	0	1	0	NA	0	7	Fair
Mahil et al.	2021	1	1	1	1	1	1	1	1	1	1	1	0	NA	0	11	Good
Rabinowitz et al.	2022	1	1	1	1	1	1	1	1	1	1	1	0	1	0	12	Good
Rubbert et al.	2021	0	1	0	0	0	1	1	1	1	0	1	0	NA	0	6	Fair
Seyahi et al.	2021	1	1	1	0	1	0	1	1	1	0	1	0	NA	1	9	Fair
Signorelli et al.	2022	1	1	1	1	1	1	1	1	1	1	1	0	NA	0	11	Good
Simon et al.	2021	1	1	1	1	1	1	NA	0	1	1	1	0	NA	1	10	Fair
Simon et al.	2022	1	1	1	0	1	1	1	1	1	0	1	0	NA	0	9	Fair
Valor-Mendez et al.	2021	1	0	0	0	0	1	NA	0	1	1	1	0	NA	0	5	Fair
Veestra et al.	2021	1	0	0	0	0	1	NA	1	0	1	1	0	NA	0	5	Fair
Vollenberg et al.	2022	1	1	1	1	1	1	1	1	1	1	1	0	1	0	12	Good
Wang et al.	2022	1	1	1	0	1	1	1	0	1	0	1	0	NA	0	8	Fair
Wong et al.	2021	1	1	1	1	1	0	NA	1	1	1	1	0	NA	0	9	Fair
Achiron et al. (1)	2021	1	1	1	1	1	0	1	1	1	1	1	0	NA	1	11	Good
Achiron et al. (3)	2022	1	1	1	1	1	NA	1	0	1	1	1	0	NA	0	9	Fair
Balcells et al.	2022	1	1	1	1	1	0	1	1	1	0	1	0	0	1	10	Fair
Barrios et.al	2022	1	1	1	1	1	1	1	1	1	1	1	0	0	0	11	Good
Ben-Tov et al.	2021	1	1	1	1	1	0	1	1	1	0	1	0	NA	1	10	Fair
Benucci et al. (1)	2022	1	1	1	1	1	1	1	0	0	0	0	0	NA	0	7	Fair
Benucci et al.	2022	1	1	1	0	1	1	1	0	0	1	0	0	NA	0	7	Fair
Bergman et al.	2022	1	1	0	0	1	1	1	1	1	1	1	0	NA	0	9	Fair
Bitoun et al.	2022	1	1	1	0	0	1	1	0	1	0	1	0	NA	0	7	Fair
Bitzenhofer et al.	2022	1	1	1	0	1	NA	1	1	1	1	1	0	NA	0	9	Fair
Boekel et al. (3)	2022	1	1	1	1	1	0	1	1	1	1	1	0	1	1	12	Good
Bonfá et al.	2022	0	0	1	0	0	0	1	0	0	1	0	0	NA	0	3	Fair
Brill et al.	2021	1	1	1	0	1	0	1	1	1	1	1	0	NA	0	9	Fair
Brill et al. (1)	2022	1	1	1	1	1	0	1	0	1	1	1	0	1	0	10	Fair
Brill et al. (2)	2022	1	1	1	1	1	1	1	1	1	1	1	0	NA	0	11	Good
Brill et al.	2022	1	0	1	0	1	0	1	1	1	1	1	0	NA	0	8	Fair
Bsteh et al.	2022	1	1	1	1	1	1	1	1	1	1	1	0	0	0	11	Good
Hadi et al.	2021	0	1	1	1	1	0	0	0	0	0	0	0	1	0	5	Fair
Khan et al.	2021	1	0	1	1	1	NA	NA	0	1	0	1	0	1	1	8	Fair
Classen et al.	2021	1	1	1	0	1	0	1	1	1	1	1	0	NA	1	10	Fair
Shehab et al.	2021	1	1	1	1	1	0	1	1	1	1	1	0	NA	0	10	Fair
Caldera et al.	2021	1	1	1	1	1	1	1	0	NA	0	NA	0	NA	0	7	Fair
Reuken et al.	2021	1	0	1	0	1	0	NA	1	1	0	1	0	NA	0	6	Fair
Zhao et al.	2022	1	1	0	1	1	1	1	0	1	0	1	0	NA	0	8	Fair
Zacharopoulou et al.	2022	1	1	1	0	1	0	1	0	1	1	1	0	NA	0	8	Fair
Yuki et al.	2022	1	1	1	1	1	1	1	1	NA	1	NA	0	NA	0	9	Fair
Lev-Tzion et al.	2022	1	1	1	0	1	0	1	0	NA	0	NA	0	1	1	7	Fair
Medeiros-Ribeiro et al.	2022	1	1	1	1	1	0	1	1	1	1	1	0	NA	1	11	Good
Pasoto et al.	2022	1	1	1	1	1	1	1	1	1	0	1	0	NA	0	10	Fair
Sampaio Barros et al.	2021	1	1	1	1	1	1	1	1	NA	0	NA	0	NA	0	8	Fair
Fabris et al.	2022	1	1	1	0	1	1	1	1	1	0	1	0	0	0	9	Fair
Tran et al.	2022	1	1	1	1	1	1	1	0	1	0	1	0	NA	0	9	Fair
Udaondo et al.	2021	1	1	1	1	1	1	1	0	1	0	1	0	NA	0	9	Fair
Verstappen et al.	2022	1	1	1	1	1	1	1	1	1	0	1	0	NA	0	10	Fair
Firinu et al.	2022	1	1	1	1	1	1	1	0	1	1	1	0	NA	0	10	Fair
Furer et al.	2022	1	1	1	1	1	0	1	1	1	0	1	0	NA	0	9	Fair
Gallo et al.	2021	1	1	0	0	0	1	1	0	1	0	1	0	NA	0	6	Fair
Jyssum et al.	2022	1	1	1	1	1	1	1	0	1	0	1	0	NA	0	9	Fair
Kim et al.	2022	1	1	1	0	1	0	1	1	1	0	1	0	1	0	9	Fair
Kornek et al.	2022	1	1	1	1	1	1	1	1	1	1	1	0	NA	1	12	Good
Krbot Skoeic et al.	2022	1	1	1	0	0	0	1	0	1	0	1	0	NA	0	6	Fair
Mauro et al.	2022	1	1	1	1	1	0	1	0	1	0	1	0	NA	1	9	Fair
Picchiant-Diamanti et al.	2021	1	1	1	1	1	1	1	1	1	0	1	0	NA	0	10	Fair
Shields et al.	2022	1	0	0	0	0	0	1	0	1	0	1	0	NA	0	4	Fair
Simader et al.	2021	1	1	1	0	1	0	1	1	1	1	1	0	NA	0	9	Fair
Stefanski et al.	2022	1	1	1	0	1	NA	1	0	1	0	1	0	NA	0	7	Fair
Medeiros-Ribeiro et al.	2021	1	1	1	1	NA	1	1	1	1	0	1	0	1	1	11	Good
Ozdede et al.	2022	1	1	1	1	1	0	1	1	1	0	1	0	NA	1	10	Fair
Pellicano et al.	2022	1	1	1	1	1	NA	1	1	0	1	0	0	NA	0	8	Fair
Sugihara et al.	2022	1	1	1	0	1	1	1	1	1	0	1	0	NA	0	9	Fair
Syversen et al.	2022	1	1	NA	1	1	NA	1	1	1	0	1	0	NA	1	9	Fair
Haidar et al.	2022	1	1	1	1	1	0	1	1	1	0	1	0	NA	1	10	Fair
Bergman et al.	2021	1	1	1	1	1	1	1	1	1	1	1	0	1	0	12	Good
Calderna et al.	2022	1	0	1	1	1	NA	1	1	1	0	1	0	NA	0	8	Fair
Capuano et al.	2022	1	1	0	1	1	1	1	1	NA	1	NA	0	1	0	9	Fair
Chen et al.	2022	1	1	1	0	1	NA	1	1	1	0	1	0	NA	1	9	Fair
Delvino et al.	2021	0	1	1	1	0	NA	1	1	1	0	1	0	NA	0	7	Fair
Doherty et al.	2022	1	1	1	1	1	1	1	0	1	0	1	0	1	0	10	Fair
Duengelhoef et al.	2022	0	1	1	1	1	1	1	1	1	1	1	0	1	0	11	Good
Ferri et al.	2022	1	1	1	0	1	1	1	1	1	1	1	0	1	0	11	Good
Firinu et al. (1)	2022	1	1	1	1	1	0	1	1	1	1	1	0	1	0	11	Good
Giannoccaro et al.	2022	1	1	1	1	1	1	1	1	1	0	1	1	NA	1	12	Good
Giossi et al.	2022	1	1	1	1	1	0	1	0	1	0	1	0	NA	0	8	Fair
Shenoy et al.	2021	1	1	1	0	1	0	1	1	0	1	1	0	NA	0	8	Fair

*Note:* Item 1. Was the research question or objective in this paper clearly stated?; Item 2. Was the study population clearly specified and defined?; Item 3. Was the participation rate of eligible persons at least 50%?; Item 4. Were all the subjects selected or recruited from the same or similar populations (including the same time period)? Were inclusion and exclusion criteria for being in the study prespecified and applied uniformly to all participants?; Item 5. Was a sample size justification, power description, or variance and effect estimates provided?; Item 6. For the analyses in this paper, were the exposure(s) of interest measured prior to the outcome(s) being measured?; Item 7. Was the timeframe sufficient so that one could reasonably expect to see an association between exposure and outcome if it existed?; Item8. For exposures that can vary in amount or level, did the study examine different levels of the exposure as related to the outcome (e.g. categories of exposure, or exposure measured as continuous variable)?; Item 9. Were the exposure measures (independent variables) clearly defined, valid, reliable, and implemented consistently across all study participants?; Item 10. Was the exposure(s) assessed more than once over time?; Item 11. Were the outcome measures (dependent variables) clearly defined, valid, reliable, and implemented consistently across all study participants?; Item 12. Were the outcome assessors blinded to the exposure status of participants?; Item 13. Was loss to follow-up after baseline 20% or less?; Item 14. Were key potential confounding variables measured and adjusted statistically for their impact on the relationship between exposure(s) and outcome(s)?; NA: Not available.

## Meta-analysis

### Serological response post-SARS-CoV-2 vaccination

**Figure 2.**
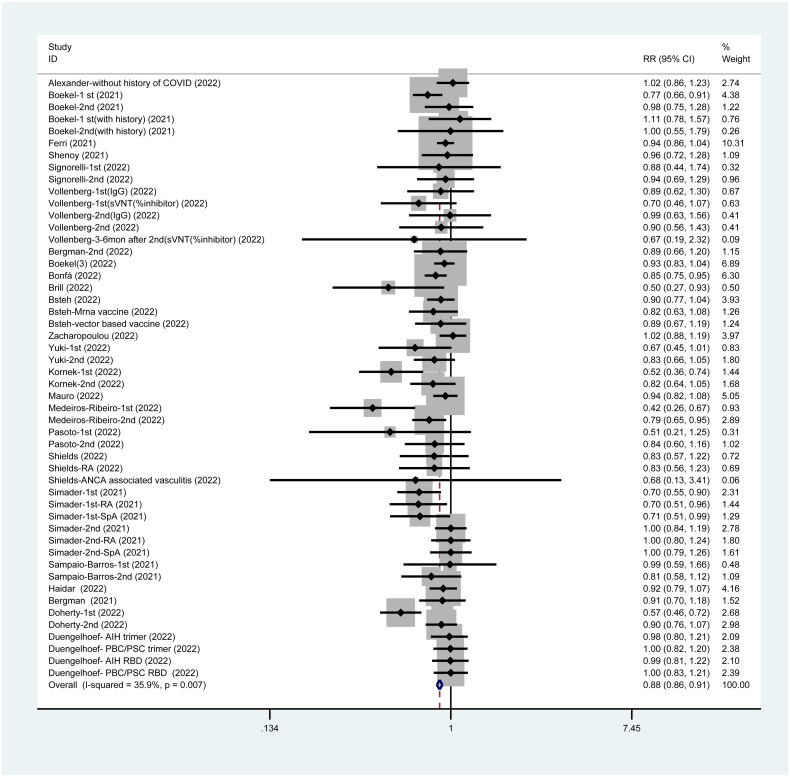
Serological response post SARS-CoV-2 vaccines.

A total of 24 studies have reported a serological response rate of 88% (95%CI: 0.86–0.91) in patients who are immunocompromised (Figure 2).

### Antibody response post-SARS-CoV-2 vaccination

**Figure 3.**
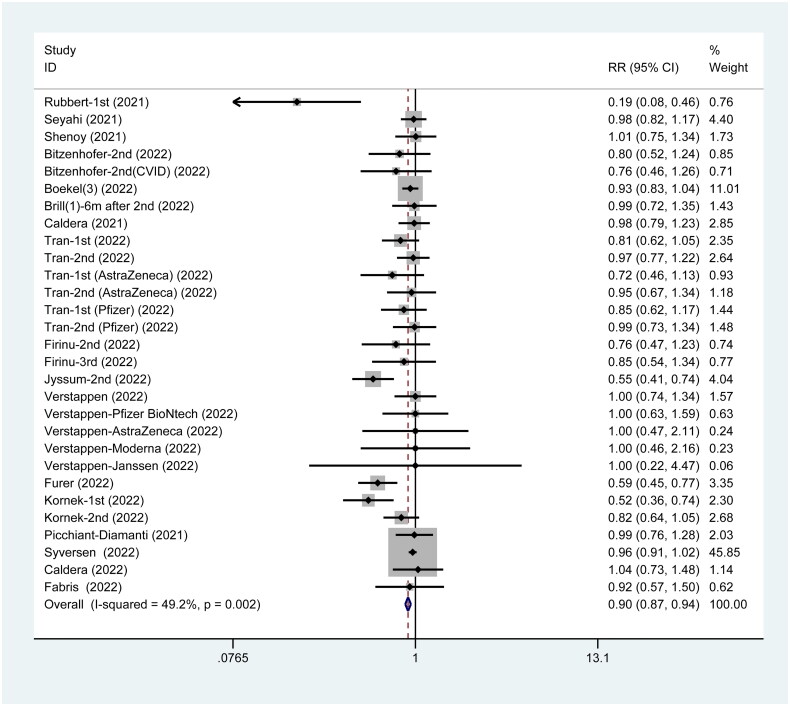
Antibody response post SARS-CoV-2 vaccines.

Among the 24 studies, 17 studies provided data on the incidence of antibody response when patients who are immunocompromised received SARS-CoV-2 vaccines. The incidence of antibody response was lower in such patients than in their healthy counterparts (RR = 0.90, 95%CI: 0.87 to 0.94) (Figure 3).

### Incidence of seropositive IgG post-SARS-CoV-2 vaccination

**Figure 4.**
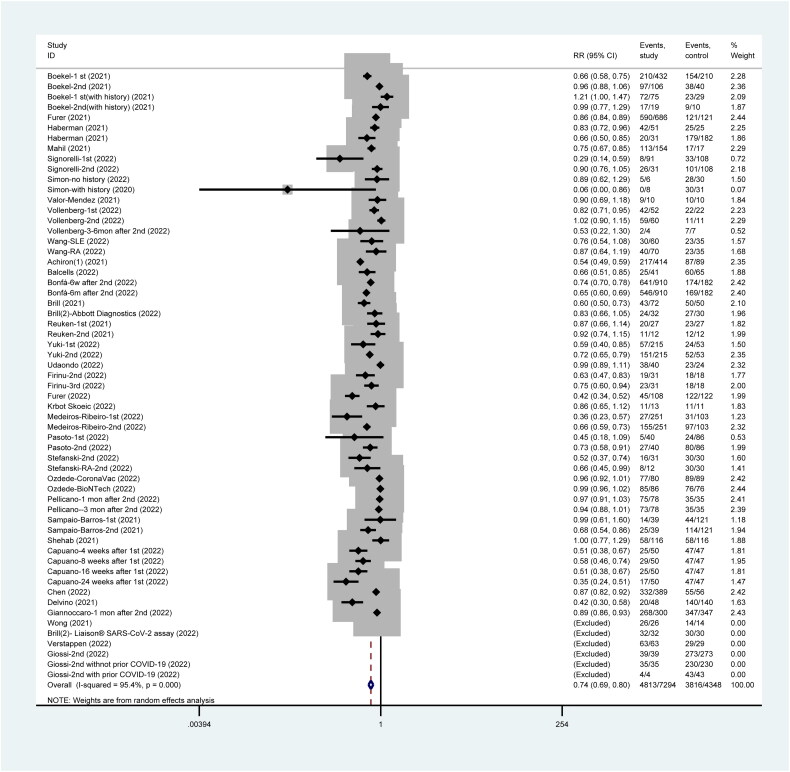
Incidence of seropositive IgG post SARS-CoV-2 vaccines.

Over 34 studies have reported on the incidence of seropositive IgG after vaccination and significant difference between patients who are immunocompromised and the healthy population (RR = 0.74, 95%CI: 0.69 to 0.80). Of the studies, four have reported producing detectable IgG titer level in both groups (Figure 4).

### Incidence of seropositive NAb post-SARS-CoV-2 vaccination

**Figure 5.**
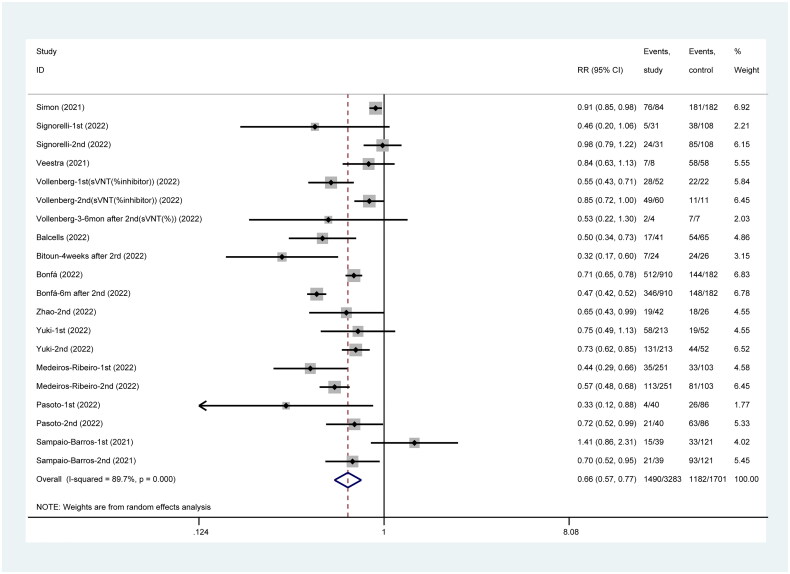
Incidence of seropositive NAb post SARS-CoV-2 vaccines.

A total of 12 published studies have reported detectable seropositive NAb titer level when participants received SARS-CoV-2 vaccines, and the pooled incidence of NAb was significantly lower in the healthy group than in vaccinated patients (RR = 0.66, 95%CI: 0.57–0.77) (Figure 5).

### Antibody titer post-SARS-CoV-2 vaccination

This meta-analysis pooled 20 studies reporting various units of antibody titer post-vaccinations and seven articles reporting significant difference in antibody titer with units (U)/ml between patients who are immunocompromised (SMD= −11.23, 95%CI: −13.47 to −9.00) and the healthy group.

**Figure 6.**
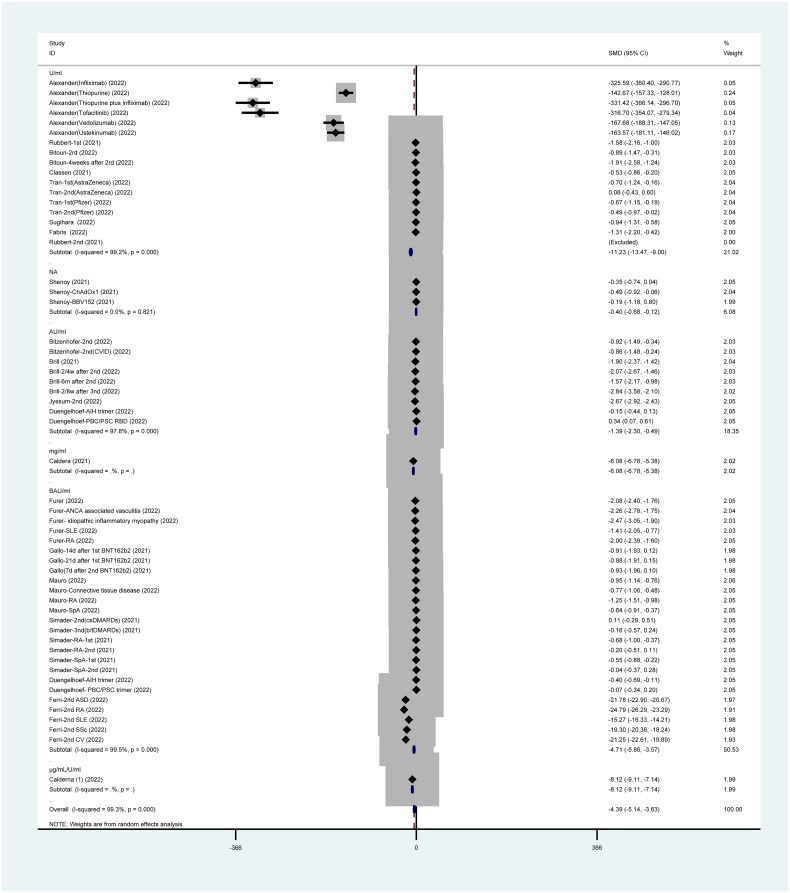
Antibody titer post SARS-CoV-2 vaccines.

Regarding antibody titer on arbitrary units (AU)/ml, only five studies have reported that antibody titer in patients who are immunocompromised was slightly lower than that in the healthy group (SMD= −1.39, 95%CI: −2.30 to −0.49). Similarly, the result of the antibody titer in BAU/ml was significantly different between the two groups (SMD= −4.71, 95%CI: −5.86 to −3.57) as reported by six studies. Moreover, other units of antibody titer post-vaccination also showed the same result such as AU/ml, that is, patients who are immunocompromised generated lower antibody titer than the healthy group (one study with mg/ml: SMD= −6.08, 95%CI: −6.78 to −5.38; one study with μg/ml: SMD= −8.12, 95%CI: −9.11 to −7.14; and one study did not indicate the unit: SMD= −0.40, 95%CI: −0.68 to −0.12). All results are presented in Figure 6.

### IgG titer post-SARS-CoV-2 vaccination

**Figure 7.**
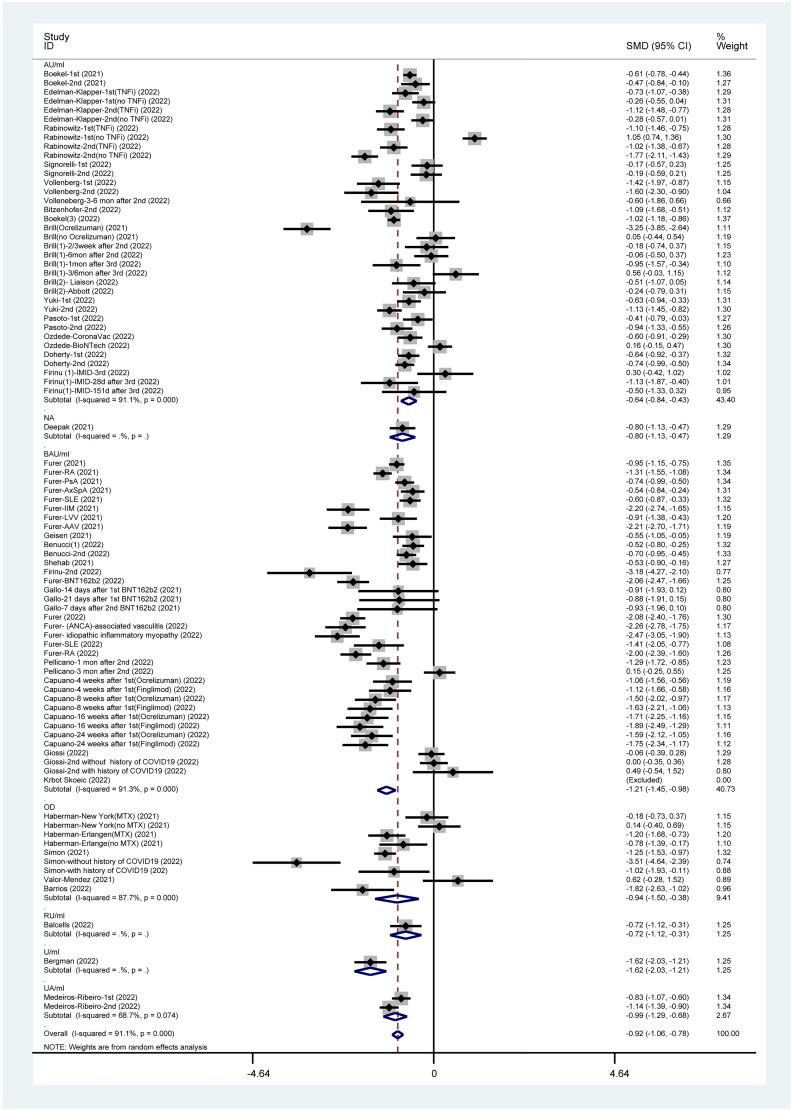
IgG Titer post SARS-CoV-2 vaccines.

Various units were used for IgG titer level testing post-vaccination, such as AU/ml, binding antibody units (BAU)/ml, U/ml, UA/ml, and optical density (OD). However, this meta-analysis identified significant differences between patients who are immunocompromised and the healthy group (pooled IgG titer: SMD= −0.92, 95%CI: −1.06 to −0.78). Many studies used AU/ml or BAU/ml as a unit of IgG titer level to measure the response to SARS-CoV-2 vaccines and presented similar results (15 studies with AU/ml: SMD= −0.64 95%CI: −0.84 to −0.43 and 12 studies with BAU/ml: SMD= −1.21, 95%CI: −1.45 to −0.98). Meanwhile, other units for IgG titer level were used by several studies. Four published articles using OD have reported slightly significant difference between patients who are immunocompromised and healthy group (SMD= −0.94 95%CI: −1.50 to −0.38) in Figure 7.

### NAb inhibitor titer post-SARS-CoV-2 vaccination

**Figure 8.**
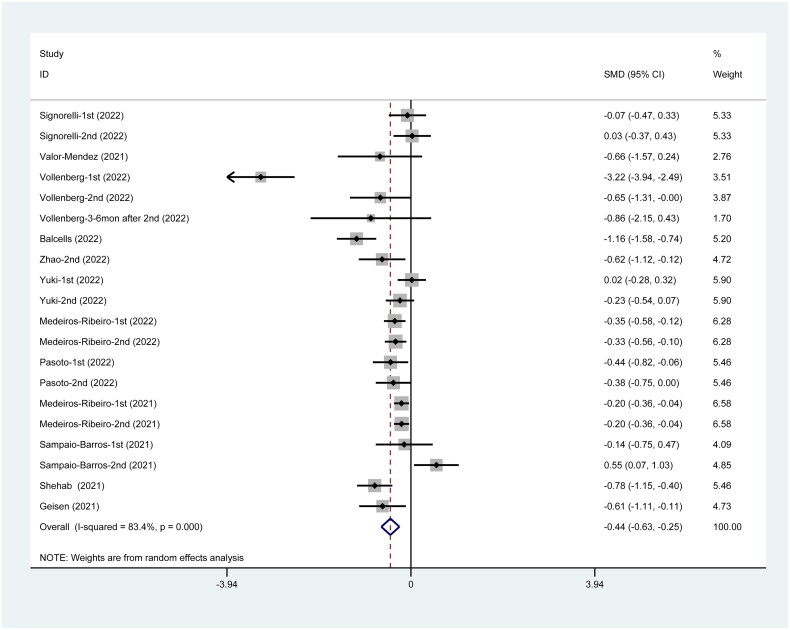
NAb Inhibitor titer post SARS-CoV-2 vaccines.

Overall, the pooled RR for NAb inhibitor titer following vaccination in 12 studies was −0.44 (95%CI: −0.63 to −0.25), which indicates that the healthy group responds to SARS-CoV-2 vaccines on a higher NAb inhibitor level than vaccinated patients. In addition, these indicators were analyzed further in vaccinated patients who are immunocompromised (Figure 8).

### Breakthrough infection post-SARS-CoV-2 vaccination

**Figure 9.**
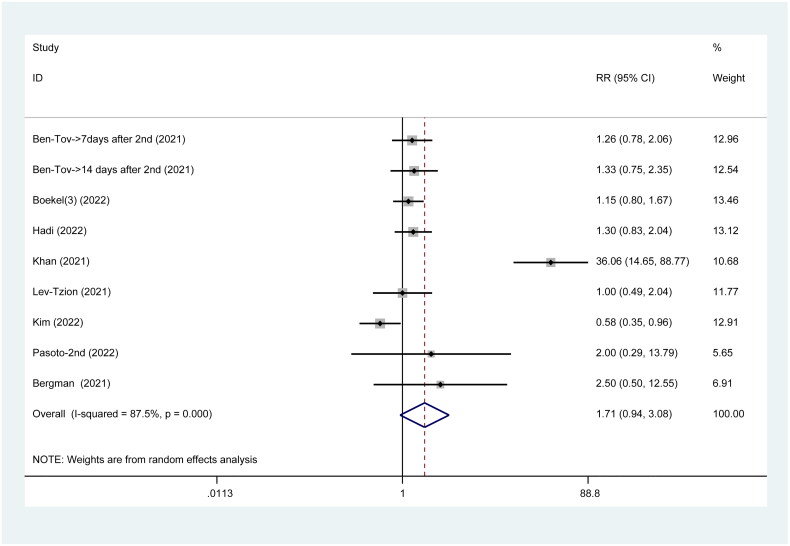
Breakthrough infection post SARS-CoV-2 vaccines.

Only eight studies have reported on the incidence of breakthrough infection after vaccination, and no significant difference was observed between the two groups (RR = 1.71, 95%CI: 0.94–3.08) (Figure 9).

## Subgroup analysis by potential factor

### Subgroup analysis of diseases of patients who are immunocompromised

The subgroup analyses involved up to 20 various immunocompromised diseases. In particular, patients with CVID, ARD, systemic lupus erythematosus (SLE), rheumatic disease (RD), rheumatoid arthritis (RA), spondyloarthritis, autoimmune conditions, and primary immunodeficiency (PID) demonstrated a slightly lower response rate than the healthy group. Significant differences in other diseases were not observed between the two groups. This may be attributable to patients with different diseases having considerably varying reactions in terms of effective and sufficient antibodies after receiving SARS-CoV-2 vaccines. Furthermore, more details of this subgroup analysis are presented in Tables 3 and 4. More patients in the seven disease groups had a comparable antibody response with healthy people. The lower seropositive IgG incidence was in those diagnosed with RD, ARD, CVID, IBD, IMID, multiple sclerosis (MS), psoriasis, and SLE (RR = 0.84, 0.89, 0.68, 0.71, 0.92, 0.82, 0.58 0.75, and 0.71, respectively). However, patients with ten diseases reported in 19 studies produced non-differential IgG against SARS-CoV-2. Regarding different units of measuring antibody or IgG titer level in the serum samples of participants, this meta-analysis conducted subgroup analysis by vital factor. Specifically, up to 20 disease types were analyzed, and patients had lower antibody titer level in their serum samples, but only in those patients with autoimmune hepatitis (AIH) for AU/ml, primary biliary cholangitis (PBC)/primary sclerosing cholangitis (PSC) for BAU/ml, and autoimmune rheumatic disease (AIRD) for OU, with SMDs of −0.15, −0.07, and −0.35, respectively.

**Table 3.** Subgroup analysis by potential factors.

Subgroup analysis	Item	Serological response	Antibody response	The incidence of seropositive IgG	The incidence of seropositive NAb	Breakthrough infection
Types of disease	IBD	0.82 (0.68, 1.00)	0.70 (0.38, 1.28)	0.93 (0.81, 1.13)	0.66 (0.43, 1.02)	NA
	IMID	0.83 (0.64, 1.06)	0.96 (0.91, 1.00)	0.82 (0.73, 0.92)	0.91 (0.85, 0.97)	NA
	ASD	0.89 (0.86, 0.93)	NA	NA	NA	NA
	AIRD	0.93 (0.85, 1.01)	NA	0.62 (0.48, 0.80)	NA	NA
	PAPS	0.89 (0.76, 1.05)	NA	0.71 (0.53, 0.96)	0.91 (0.85, 0.97)	NA
	CVID	0.80 (0.69, 0.94)	0.76 (0.46, 1.26)	0.83 (0.48, 1.43)	NA	NA
	ARD	0.63 (0.51, 0.78)	NA	0.80 (0.75, 0.86)	0.55 (0.43, 0.71)	NA
	MS	0.54 (0.18, 1.59)	0.99 (0.72, 1.35)	0.76 (0.69, 0.84)	NA	NA
	SLE	0.68 (0.53, 0.87)	NA	0.79 (0.66, 0.95)	0.73 (0.63, 0.84)	NA
	Neuroimmunologic disease	0.50 (0.23, 1.09)	0.66 (0.42, 1.05)	NA	NA	NA
	RD	0.89 (0.85, 0.93)	NA	0.91 (0.81, 1.02)	0.50 (0.34, 0.73)	NA
	pSS	0.66 (0.44, 1.01)	1.00 (0.74, 1.34)	0.86 (0.68, 1.07)	0.55 (0.25, 1.20)	NA
	RA	0.62 (0.46, 0.85)	0.74 (0.41, 1.34)	NA	0.65 (0.43, 0.99)	NA
	ANCA	0.51 (0.13, 2.06)	NA	0.94 (0.84, 1.06)	NA	NA
	SpA	0.55 (0.42, 0.72)	NA	NA	NA	NA
	SSc	0.77 (0.54, 1.11)	NA	0.93 (0.79, 1.09)	0.97 (0.49, 1.93)	NA
	Autoimmune condition	0.86 (0.79, 0.92)	NA	NA	0.32 (0.17, 0.60)	NA
	PID	0.85 (0.72, 0.99)	NA	NA	NA	NA
	AIH	0.97 (0.93, 1.01)	NA	NA	NA	NA
	PBC/PSC	0.99 (0.97, 1.02)	NA	NA	NA	NA
	AIDs	NA	0.81 (0.55,1.18)	0.62 (0.48, 0.80)	NA	NA
	GCA	NA	NA	0.81 (0.66, 1.00)	NA	NA
	Patients on RTX	NA	NA	0.56 (0.26, 1.18)	NA	NA
	Psoriasis	NA	NA	0.85 (0.59, 1.22)	NA	NA
	BS	NA	NA	0.99 (0.55, 1.55)	NA	NA
Treatment	No treatment	0.97 (0.91, 1.04)	0.98 (0.97, 1.00)	1.02 (0.97, 1.06)	NA	NA
	IFX	0.81 (0.56, 1.18)	NA	0.69 (0.51, 0.93)	0.53 (0.19, 1.48)	NA
	Usterkinumab	1.01 (0.87, 1.16)	NA	0.76 (0.53, 1.11)	0.85 (0.69, 1.05)	NA
	Vedolizumab	0.98 (0.80, 1.21)	NA	0.95 (0.80, 1.14)	0.76 (0.43, 1.33)	NA
	Tofacitinib	1.07 (0.98, 1.17)	NA	NA	NA	NA
	Prednisone	0.85 (0.66, 1.09)	NA	0.93 (0.70, 1.23)	NA	NA
	TNFi monotherapy	0.99 (0.82, 1.18)	NA	0.97 (0.88, 1.07)	NA	NA
	MTX	0.74 (0.40, 1.38)	0.98 (0.86, 1.12)	0.56 (0.33, 0.95)	0.90 (0.76, 1.07)	NA
	Monotherapy	0.70 (0.28, 1.72)	NA	0.70 (0.28, 1.72)	NA	NA
	anti-TNF and methotrexate	0.66 (0.23, 1.92)	NA	NA	NA	NA
	anti-CD20 therapy	0.38 (0.15, 0.92)	0.54 (0.40, 0.74)	0.46 (0.34, 0.62)	NA	NA
	Sulfasalazine	0.83 (0.30, 2.29)	NA	NA	NA	NA
	LEF	0.91 (0.73, 1.13)	NA	NA	NA	NA
	HCQ	0.97 (0.90, 1.05)	NA	NA	NA	NA
	ADA	0.55 (0.17, 1.74)	NA	0.81 (0.60, 1.09)	0.64 (0.47, 0.87)	NA
	RTX	NA	0.47 (0.36, 0.59)	NA	0.32 (0.17, 0.58)	NA
	tsDMARDs	NA	0.67 (0.19, 2.35)	NA	NA	NA
	cDMARDs	NA	0.88 (0.76, 1.02)	NA	NA	NA
	Immunomodulator	NA	NA	0.88 (0.78, 0.98)	NA	NA
	Fingolimod	NA	NA	0.37 (0.26, 0.51)	NA	NA
	Ocrelizumab	NA	NA	0.57 (0.24, 1.38)	NA	NA
	IL-17i	NA	NA	0.97 (0.92, 1.02)	NA	NA
	Combined therapy	NA	NA	0.59 (0.31, 1.11)	NA	NA
Immunoassay	ECLIA	0.86 (0.79, 0.94)	0.83 (0.74, 0.93)	0.92 (0.86, 0.99)	NA	NA
	ELISA	0.89 (0.84, 0.95)	0.96 (0.89, 1.04)	0.73 (0.64, 0.83)	0.70 (0.60, 0.82)	NA
	CLIA	0.86 (0.81, 0.91)	0.83 (0.74, 0.92)	0.69 (0.61, 0.78)	0.32 (0.17, 0.60)	NA
	ECLIA and CLIA	0.99 (0.86, 1.14)	NA	NA	NA	NA
	Beckman Coulter	0.92 (0.79, 1.07)	NA	NA	NA	NA
	Flow Cytometry	NA	0.55 (0.41, 0.74)	NA	NA	NA
	CMIA	NA	NA	0.82 (0.65, 1.03)	NA	NA
	Indirect CLIA and MIA	NA	1.00 (0.74, 1.34)	NA	NA	NA
	NR	0.85 (0.75, 0.95)	0.96 (0.91, 1.02)	0.69 (0.61, 0.79)	0.58 (0.38, 0.87)	NA
History of SARS-CoV-2 infection	With the history of infection	0.79 (0.70, 0.89)	0.62 (0.47, 0.84)	0.87 (0.76, 1.00)	0.91 (0.75, 1.09)	1.15 (0.80, 1.67)
	Without the history of infection	0.82 (0.75, 0.90)	0.95 (0.88, 1.02)	0.71 (0.63, 0.80)	0.68 (0.57, 0.81)	1.33 (1.01, 1.76)
	Unknow	0.62 (0.47, 0.82)	0.74 (0.60, 0.92)	0.70 (0.62, 0.79)	0.58 (0.46, 0.74)	0.72 (0.48, 1.08)
Vaccine dose	1st dose	0.73 (0.67, 0.79)	0.60 (0.49, 0.73)	0.73 (0.66, 0.79)	0.62 (0.43, 0.89)	NA
	2nd dose	0.92 (0.89, 0.96)	0.93 (0.89, 0.97)	0.87 (0.83, 0.90)	0.68 (0.57, 0.80)	1.28 (0.99, 1.66)
	3rd dose	0.50 (0.27, 0.93)	0.85 (0.54, 1.34)	0.84 (0.59, 1.19)	NA	0.94 (0.70, 1.26)
	NA	0.83 (0.57, 1.22)	NA	NA	NA	NA
Vaccine type	Adenoviral vector vaccine	0.81 (0.70, 0.94)	0.75 (0.39, 1.41)	0.89 (0.84, 0.95)	NA	NA
	Inactivated virus and adenoviral vector vaccine	0.93 (0.85, 1.01)	1.01 (0.89, 1.15)	NA	NA	NA
	Inactivated virus vaccine	0.71 (0.65, 0.78)	0.95 (0.91, 1.00)	0.70 (0.62, 0.80)	0.79 (0.67, 0.94)	NA
	mRNA and adenoviral vector vaccine	0.81 (0.72, 0.92)	0.85 (0.76, 0.94)	0.78 (0.59, 1.02)	NA	NA
	mRNA vaccine	0.76 (0.68, 0.86)	0.71 (0.58, 0.86)	0.72 (0.65, 0.80)	0.63 (0.54, 0.74)	NA
	NR	NA	NA	0.86 (0.65, 1.12)	0.32 (0.17, 0.60)	NA
Vaccine subtype	Pfizer, Moderna and Janssen	0.96 (0.86, 1.08)	NA	NA	NA	NA
	Pfizer, Moderna, AstraZeneca, Janssen	0.87 (0.82, 0.93)	0.93 (0.82, 1.05)	0.80 (0.49, 1.30)	NA	0.97 (0.72, 1.30)
	Pfizer and Moderna	0.91 (0.85, 0.98)	0.61 (0.38, 0.96)	0.88 (0.68, 1.14)	0.80 (0.61, 1.05)	1.30 (0.83, 2.03)
	BBV152 and AstraZeneca	0.96 (0.72, 1.28)	1.01 (0.89, 1.15)	NA	NA	NA
	Sinovac-CoronaVac	0.80 (0.74, 0.87)	0.95 (0.91, 1.00)	0.70 (0.62, 0.80)	0.73 (0.67, 0.79)	1.96 (0.28, 13.54)
	Pfizer	0.90 (0.81, 1.01)	0.76 (0.59, 0.98)	0.72 (0.65, 0.80)	0.92 (0.78, 1.09)	1.26 (0.91, 1.73)
	Vector-based vaccine	0.89 (0.67, 1.19)	NA	NA	NA	NA
	Pfizer and AstraZeneca	0.83 (0.57, 1.22)	NA	0.76 (0.53, 1.08)	NA	NA
	AstraZeneca	NA	0.75 (0.39, 1.41)	0.89 (0.84, 0.95)	NA	NA
	Pfizer, Moderna and AstraZeneca	NA	0.60 (0.16, 2.26)	NA	NA	NA
	Moderna	NA	NA	0.53 (0.46, 0.60)	NA	NA
	NR	NA	NA	0.86 (0.65, 1.12)	0.47 (0.23, 0.96)	NA
Variant of concerns	Delta (B.1.617.2) variant	0.87 (0.85, 0.89)	0.73 (0.55, 0.96)	NA	NA	1.15 (0.80, 1.67)
	B.1.1.7 variant	NA	NA	0.75 (0.67, 0.85)	NA	NA
	None	0.77 (0.71, 0.83)	0.80 (0.73, 0.87)	0.73 (0.68, 0.79)	0.66 (0.57, 0.77)	1.28 (0.99, 1.66)

*Note:* mRNA, messenger RNA; pSS, Primary Sjögren’s syndrome; IgG, immunoglobin G; IFX, infliximab; ADA: adalimumab; anti-CD20, CD-20 inhibitors; IL17i, interleukin 17 inhibitors; MTX, methotrexate; TNFi, tumour necrosis factor inhibitors; HCQ, hydroxychloroquine; LEF, leflunomide; bDMARD, biological disease-modifying anti-rheumatic drug; csDMARD, conventional synthetic disease-modifying anti-rheumatic drug; RTX, rituximab; PAPS, primary antiphospholipid syndrome; PSC, primary sclerosing cholangitis; PBC, primary biliary cholangitis; MS: multiple sclerosis; CVID, Common Variable Immunodeficiency; AIH, autoimmune hepatitis; AID, autoinflammatory diseases; ARD, autoimmune rheumatic diseases; ASD, autoimmune systemic diseases; RA, Rheumatoid arthritis; RD, rheumatic disease; IMID, immune-mediated inflammatory disease; AIIRD, autoimmune inflammatory rheumatic diseases; SLE, systemic lupus erythematosus; PID, primary immunodeficiency; IBD, inflammatory bowel disease; GCA, giant cell arteritis; BS, Behçet’s syndrome; SpA, spondyloarthritis; SSc, systemic sclerosis; ANCA, antineutrophil cytoplasmic antibody; CMIA, chemiluminescent immunoassay microparticles; MIA, luminescent immunoassay microparticles; CLIA, chemiluminescent immunoassay; ELISA, Enzyme-linked immunosorbent assay; ECLIA, electrochemiluminescence immunoassay analyzer; NA: not available.

**Table 4.** Result of subgroup analysis about continue data by potential factors.

Subgroup analysis	Unit	Item	Antibody titer	IgG titer
Types of disease	AU/ml	AIH	−0.15 (−0.44, 0.13)	NA
		IBD	NA	−0.63 (−0.71, −0.55)
		Autoimmune disease	NA	−1.09 (−1.68, −0.51)
		BS	NA	−0.22 (−0.44, 0.00)
		IMID	NA	−0.95 (−1.10, −0.80)
		PAPS	NA	−0.18 (−0.46, 0.10)
		SLE	NA	−0.88 (−1.10, −0.66)
		pSS	NA	−0.66 (−0.94, −0.39)
		ARD	NA	−4.62 (−4.81, −4.44)
		CVID	−0.86 (−1.48, −0.24)	NA
		MS	−2.00 (−2.30, −1.71)	−0.47 (−0.67, −0.28)
		PBC/PSC	0.34 (0.07, 0.61)	NA
		RA	−2.67 (−2.92, −2.43)	NA
	BAU/ml	AIH	−0.40 (−0.69, −0.11)	NA
		AAV	NA	−2.21 (−2.70, −1.71)
		ANCA	−2.26 (−2.78, −1.75)	−2.26 (−2.78, −1.75)
		Connective tissue disease	−0.77 (−1.06, −0.48)	NA
		CV	−21.25 (−22.61, −19.89)	NA
		IMID	NA	−3.18 (−4.27, −2.10)
		Idiopathic inflammatory myopathy	−2.47 (−3.05, −1.90)	−2.47 (−3.05, −1.90)
		MS	−0.91 (−1.50, −0.31)	−1.13 (−1.29, −0.96)
		PBC/PSC	−0.07 (−0.34, 0.20)	NA
		RA	−1.22 (−1.37, −1.06)	−1.18 (−1.34, −1.02)
		SLE	−5.13 (−5.68, −4.58)	−0.73 (−0.97, −0.48)
		SpA	−0.44 (−0.62, −0.26)	−0.54 (−0.84, −0.24)
		SSc	−19.30 (−20.36, −18.24)	−0.51 (−0.81, −0.22)
		PsA	NA	−0.65 (−0.83, −0.47)
		LVV	NA	−0.91 (−1.38, −0.43)
		CID	NA	−0.55 (−1.05, −0.05)
		IBD	−6.08 (−6.78, −5.38)	−0.53 (−0.90, −0.16)
	OU	AIRD	−0.35 (−0.74, 0.04)	NA
	mg/ml	IBD	−6.08 (−6.78, −5.38)	NA
	OD	AIDs	0.62 (−0.28, 1.52)	NA
		CVID	−1.82 (−2.63, −1.02)	NA
		IMID	−0.88 (−1.08, −0.69)	NA
		Patients on RTX	NA	−2.00 (−2.71, −1.30)
	U/ml	Autoimmune disease	−1.33 (−1.77, −0.89)	−0.82 (−1.21, −0.43)
		AIDs	−1.31 (−2.20, −0.42)	NA
		IBD	−0.99 (−1.27, −0.70)	NA
		IMID	−0.45 (−0.70, −0.20)	NA
		RMD	−0.94 (−1.31, −0.58)	NA
		CVID	NA	−1.62 (−2.03, −1.21)
	UA/ml	ARD	NA	−0.98 (−1.15, −0.81)
Treatment	AU/ml	Immunosuppressed treatment	−0.09 (−0.39, 0.20)	NA
		Ocrelizumab	−2.00 (−2.30, −1.71)	−3.25 (−3.85, −2.65)
		RTX	−2.67 (−2.92, −2.43)	NA
		TNFi and methotrexate	NA	−0.52 (−0.83, −0.22)
		Colchicine	NA	0.20 (−0.16, 0.56)
		cDMARDs	NA	−0.48 (−0.71, −0.26)
		No treatment	NA	−0.17 (−0.29, −0.04)
		TNFi monotherapy	NA	−0.72 (−0.85, −0.59)
		ADA	NA	−1.13 (−1.71, −0.55)
		anti-CD20 therapy	NA	−0.84 (−1.32, −0.35)
		anti-CD21 therapy	NA	−0.89 (−1.71, −0.07)
		anti-CD21 therapy	NA	−1.38(−2.22, −0.54)
		Immunomodulator	NA	0.15(−0.38, 0.68)
		Infliximab	NA	−1.29 (−1.81, −0.76)
		JAK inhibitor	NA	0.59 (−0.42, 1.59)
		Methotrexate	NA	−0.82 (−1.03, −0.61)
		Methotrexate Monotherapy	NA	−0.71 (−0.97, −0.44)
		Non-TNFalpha therapy	NA	−0.25(−0.40, −0.09)
		Prednisone monotherapy	NA	−0.24(−0.64, 0.16)
		Ustekinumab	NA	−0.57(−1.12, −0.02)
		Vedolizumab	NA	−0.57(−1.19, 0.04)
	BAU/ml	ABA	−1.50 (−2.04, −0.96)	NA
		Immunosuppressed treatment	−0.40 (−0.70, −0.10)	NA
		MTX	−1.27 (−1.53, −1.01)	NA
		bDMARDs/tsDMARDs	NA	−0.67(−0.93, −0.42)
		csDMARDs	NA	−0.55 (−1.08, −0.02)
		Finglimod	NA	−1.51(−1.79, −1.23)
		IFX	NA	−0.53 (−0.90, −0.16)
		JAKi	NA	−1.82 (−2.33, −1.32)
		MTX	NA	−0.58 (−1.24, 0.08)
		No treatment	NA	−0.25, (−0.54, 0.05)
		Ocrelizumab	NA	−1.43 (−1.69, −1.18)
		RTX	NA	−2.33 (−2.9, −1.74)
		TNFi	NA	−0.88 (−1.15, −0.60)
	mg/ml	Immunosuppressed treatment	−3.54 (−4.05, −3.03)	NA
	U/ml	ABA	−1.78 (−2.29, −1.27)	NA
		Continued DMARD therapy	−0.45 (−0.70, −0.20)	NA
		MTX	−1.74 (−2.24, −1.24)	NA
		RTX	−1.32 (−1.72, −0.93)	NA
		Ustekinumab	−0.30 (−0.88, 0.27)	NA
		Withhold DMARD therapy	−0.12 (−0.37, 0.14)	NA
	OD	cDMARDs	NA	−1.58 (−2.07, −1.09)
		MTX	NA	−0.76 (−1.12, −0.40)
		No treatment	NA	−1.29 (−1.73, −0.84)
		Non-MTX	NA	−0.26 (−0.67, 0.14)
		Immunomodulator	NA	−0.77 (−1.29, −0.24)
		RTX	NA	−0.89 (−1.47, −0.31)
Immunoassay	AU/ml	CLIA	−2.26 (−2.43, −2.08)	−1.07 (−1.89, −0.24)
		CLIA and ELISA	NA	−0.65 (−1.25, −0.05)
		CLIA and ECLIA	0.11 (−0.09, 0.30)	NA
		ELISA	NA	−0.78 (−1.03, −0.54)
		ECLIA	NA	−0.22 (−0.97, 0.53)
	BAU/ml	CLIA	−1.63 (−1.78, −1.47)	−1.29 (−1.65, −0.93)
		CLIA and ECLIA	−0.22 (−0.42, −0.03)	NA
		ELISA	NA	−0.54 (−0.84, −0.24)
		ECLIA	−0.02 (−0.31, 0.26)	NA
	mg/ml	ECLIA	−6.08 (−6.78, −5.38)	NA
	OU	CLIA	−0.35 (−0.74, 0.04)	NA
	U/ml	ECLIA	−1.06 (−1.25, −0.86)	−1.12 (−1.69, −0.55)
		Indirect CLIA	−0.45 (−0.70, −0.20)	NA
	UA/ml	CLIA	NA	−0.99 (−1.29, −0.68)
	OD	ELISA	NA	−0.94 (−1.50, −0.38)
	RU/ml	ELISA	NA	−0.72 (−1.12, −0.31)
History of SARS-CoV-2 infection	AU/ml	With the history of infection	−2.67 (−2.92, −2.43)	−0.68 (−0.77, −0.60)
		Without the history of infection	0.11 (−0.09, 0.30)	−1.88 (−1.99, −1.77)
		Unknow	−1.64 (−1.88, −1.40)	−0.69 (−0.79, −0.59)
	BAU/ml	With the history of infection	−0.95 (−1.14, −0.76)	−0.44 (−0.72, −0.15)
		Without the history of infection	−0.87 (−1.07, −0.68)	−1.10 (−1.26, −0.94)
		Unknow	−0.93 (−1.13, −0.72)	−0.95 (−1.07, −0.84)
	U/ml	With the history of infection	−0.53 (−0.86, −0.20)	NA
		Without the history of infection	−1.34 (−1.63, −1.05)	−1.62 (−2.03, −1.21)
		Unknow	−0.66 (−088, −0.44)	−0.82 (−1.21, −0.43)
	UA/ml	Without the history of infection	NA	−0.98 (−1.15, −0.81)
	OD	With the history of infection	NA	−0.84 (−1.06, −0.62)
		Without the history of infection	NA	−1.03 (−1.36, −0.70)
		Unknow	NA	−1.82 (−2.63, −1.02)
Vaccine dose	AU/ml	1st dose	NA	−0.93 (−1.01, −0.84)
		2nd dose	−1.14 (−1.27, −1.01)	−1.08 (−1.15, −1.01)
		3rd dose	−2.84 (−3.58, −2.10)	−0.30 (−0.60, 0.01)
	BAU/ml	1st dose	−0.64 (−0.86, −0.42)	−1.46 (−1.65, −1.28)
		2nd dose	−0.92 (−1.03, −0.80)	−0.80 (−0.90, −0.70)
	mg/ml	2nd dose	−6.08 (−6.78, −5.38)	NA
	NA	2nd dose	−0.35 (−0.74, 0.04)	−0.80 (−1.13, −0.47)
	OD	2nd dose	NA	−0.94 (−1.12, −0.76)
	RU/ml	2nd dose	NA	−0.72 (−1.12, −0.31)
	UA/ml	1st dose	NA	−0.83 (−1.07, −0.60)
		2nd dose	NA	−1.14 (−1.39, −0.90)
	U/ml	1st dose	−0.93 (−1.24, −0.63)	NA
		2nd dose	−0.79 (−0.97, −0.61)	−1.20 (−1.49, −0.92)
Vaccine type	AU/ml	mRNA vaccine	−1.27 (−2.12, −0.43)	−0.53 (−0.62, −0.44)
		mRNA and adenoviral vector vaccine	−2.67 (−2.92, −2.43)	−0.77 (−0.86, −0.67)
		Inactivated virus vaccine	NA	−1.96 (−2.06, −1.85)
	BAU/ml	mRNA vaccine	NA	−0.95 (−1.04, −0.86)
	None	inactivated virus vaccine	−0.19 (−1.18, 0.80)	NA
		mRNA vaccine	NA	−0.80 (−1.13, −0.47)
		Adenoviral vector vaccine	−0.49 (−0.92, −0.06)	NA
	OD	mRNA vaccine	NA	−0.94 (−1.12, −0.76)
	UA/ml	Inactivated virus vaccine	NA	−0.98 (−1.15, −0.81)
	U/ml	Adenoviral vector vaccine	−0.30 (−1.07, 0.46)	NA
		mRNA vaccine	−1.06 (−1.42, −0.71)	−1.20 (−1.49, −0.92)
		mRNA and adenoviral vector vaccine	−206.12 (−310.20, −102.04)	NA
	RU/ml	Inactivated virus vaccine	NA	−0.72 (−1.12, −0.31)
Vaccine subtype	AU/ml	Pfizer and Moderna	−0.19 (−0.78, 0.39)	−1.30 (−1.63, −0.96)
		Sinovac-CoronaVac	NA	−1.96 (−2.06, −1.85
		Pfizer, Moderna, AstraZeneca, Janssen	NA	−0.77 (−0.86, −0.67)
		Pfizer, Moderna, AstraZeneca	−2.67 (−2.92, −2.43)	NA
		Pfizer	−2.05 (−2.51, −1.59)	−0.48 (−0.57, −0.38)
	BAU/ml	mRNA vaccine	−0.02 (−0.31, 0.26)	NA
		NR	NA	NA
		Pfizer	−1.22 (−1.88, −0.54)	−0.97 (−1.06, −0.88)
		Pfizer and Moderna	−7.36 (−13.13, −1.59)	−0.55 (−1.05, −0.05)
	OD	Pfizer	NA	−0.94 (−1.12, −0.76)
	RU/ml	Sinovac-CoronaVac	NA	−0.72 (−1.12, −0.31)
	UA/ml	Sinovac-CoronaVac	NA	−0.98 (−1.15, −0.81)
	mg/ml	Pfizer and Moderna	−6.08 (−6.78, −5.38)	NA
	None	AstraZeneca	−0.49 (−0.92, −0.06)	NA
		Pfizer and Moderna	NA	−0.80 (−1.13, −0.47)
		BBV152	−0.19 (−1.18, 0.80)	NA
	U/ml	AstraZeneca	−0.30 (−1.07, 0.46)	NA
		Pfizer	−0.94 (−1.33, −0.54)	−1.20 (−1.49, −0.92)
		Pfizer and Moderna	−1.50 (−1.99, −1.02)	NA
		Pfizer, Moderna and Janssen	−0.53 (−0.86, −0.20)	NA
		Pfizer, Moderna, AstraZeneca	−239.56 (−301.94, −177.18)	NA
Variant of concerns	AU/ml	Delta (B.1.617.2) variant	−2.67 (−2.92, −2.43)	−1.02 (−1.18, −0.86)
		None	−0.58 (−0.74, −0.42)	−0.99 (−1.04, −0.93)
	BAU/ml	None	−0.92 (−1.03, −0.80)	−0.95 (−1.04, −0.86)
	mg/ml	None	−6.08 (−6.78, −5.38)	NA
	NA	None	−0.35 (−0.74, 0.04)	−0.80 (−1.13, −0.47)
	OD	None	NA	−0.94 (−1.12, −0.76)
	RU/ml	None	NA	−0.72 (−1.12, −0.31)
	U/ml	None	−0.83 (−0.98, −0.67)	−1.20 (−1.49, −0.92)
	UA/ml	None	NA	−0.98 (−1.15, −0.81)

*Note:* BAU, binding antibody units; RU, relative units; AU, arbitrary units, U, unit; OD, optical density; IgG, immunoglobulin G; NAb, neutralising antibody; mRNA, messenger RNA; pSS, Primary Sjögren’s syndrome; IgG, immunoglobin G; IFX, infliximab; ABA, abatacept; ADA: adalimumab; anti-CD20, CD-20 inhibitors; anti-CD21, CD-21 inhibitors IL17i, interleukin 17 inhibitors; JAKi, Janus kinase inhibitors; MTX, methotrexate; TNFi, tumour necrosis factor inhibitors; HCQ, hydroxychloroquine; LEF, leflunomide; bDMARD, biological disease-modifying anti-rheumatic drug; csDMARD, conventional synthetic disease-modifying anti-rheumatic drug; RTX, rituximab; PAPS, primary antiphospholipid syndrome; PSC, primary sclerosing cholangitis; PBC, primary biliary cholangitis; MS: multiple sclerosis; CV, cryoglobulinemic vasculitis; CVID, Common Variable Immunodeficiency; AAV, ANCA-associated vasculitis; AIH, autoimmune hepatitis; AIDs, autoinflammatory diseases; ARD, autoimmune rheumatic diseases; ASD, autoimmune systemic diseases; RA, Rheumatoid arthritis; RD, rheumatic disease; RMD, Rheumatic musculoskeletal diseases; IMID, immune-mediated inflammatory disease; AIIRD, autoimmune inflammatory rheumatic diseases; SLE, systemic lupus erythematosus; PID, primary immunodeficiency; IBD, inflammatory bowel disease; GCA, giant cell arteritis; BS, Behçet’s syndrome; SpA, spondyloarthritis; SSc, systemic sclerosis; ANCA, antineutrophil cytoplasmic antibody; CMIA, chemiluminescent immunoassay microparticles; MIA, luminescent immunoassay microparticles; CLIA, chemiluminescent immunoassay; ELISA, Enzyme-linked immunosorbent assay; ECLIA, electrochemiluminescence immunoassay analyzer; NA: not available.

### Subgroup analysis of patients receiving treatments

The subgroup of patients who received anti-CD20 therapy was distinctively smaller than the healthy group, based on pooling two cohort studies on serological response post-vaccination (RR = 0.38, 95%CI: 0.15–0.92) and one study on antibody response (RR = 0.54, 95%CI: 0.40–0.74). Nevertheless, patients on rituximab therapy demonstrated a lower antibody response at 0.47 (95%CI: 0.36–0.59). For seropositive IgG concentrations, those patients who received immunomodulator (RR = 0.88, 95%CI: 0.78, 0.98), anti-CD20 therapy (RR = 0.46, 95%CI: 0.34, 0.62), methotrexate (RR = 0.56, 95%CI: 0.33, 0.95), and infliximab (RR = 0.69, 95%CI: 0.51, 0.93) failed to reach comparable IgG level with that of the healthy group. Patients administered adalimumab (RR = 0.64, 95%CI: 0.47, 0.87) and rituximab (RR = 0.32, 95%CI: 0.17, 0.58) reported decreased NAb incidence compared to those who received infliximab, ustekinumab, methotrexate, or vedolizumab. Many patients who are immunocompromised vaccinated against SARS-CoV-2 during continuous drug treatment had similar detectable serological antibody level with that of the healthy group, and more details of the subgroup analysis are presented in Table 3. For continuous data of SARS-CoV-2 antibody titer level, only patients with ustekinumab (SMD= −0.30 (U/ml, 95%CI: −0.88 to 0.27) did not have significantly reduced antibody titer level. However, patients receiving ocrelizumab (SMD= −2.00 AU/ml, 95%CI: −2.30 to −1.71), rituximab (SMD= −2.67 AU/ml, 95%CI: −2.92 to −2.43; SMD= −1.32 U/ml, 95%CI: −1.72 to −0.93), abatacept (SMD= −1.50 BAU/ml, 95%CI: −2.04 to −0.96; SMD= −1.78 U/ml, 95%CI: −2.29 to −1.27), methotrexate (SMD= −1.27 BAU/ml, 95%CI: −1.53 to −1.01; SMD= −1.74 U/ml, 95%CI: −2.24 to −1.24), and continued disease-modifying anti-rheumatic drug (DMARD) therapy (SMD= −0.45 U/ml, 95%CI: −0.70 to −0.20) were not significantly affected, thus suggesting that patients who are immunocompromised could not develop a similar antibody titer level with that of the healthy group. Thus, antibody titer level in patients who are immunocompromised differed depending on drug therapy administration at the time of vaccination (Table 4).

IgG titer level have differences based on immunosuppressive drugs, which many of those illustrated decreased titer in patients who are immunocompromised compared to that in the healthy group. Specifically, IgG titer level were reduced in patients who are immunocompromised and received anti-TNF and methotrexate after the first and second vaccinations compared to the general and sufficient titer leves in the healthy population (SMD= −0.52 AU/ml, 95%CI: −0.83 to −0.22). Ozdede et al. have reported that the titer level of BioNTech or CoronaVac recipients was not significantly different between subgroups of patients on colchicine and the healthy group (SMD = 0.20 AU/ml, 95%CI: −0.16 to 0.56). In addition to combined treatment of anti-TNF and methotrexate, patients on twelve drugs demonstrated significant difference including csDMARDs therapy (SMD= −0.48 AU/ml, 95%CI: −0.71 to −0.26), TNF inhibitor monotherapy (SMD= −0.72 AU/ml, 95%CI: −0.85 to −0.59), adalimumab (SMD= −1.13 AU/ml, 95%CI: −1.71 to −0.55), anti-CD20 therapy (SMD= −0.84 AU/ml, 95%CI: −1.32 to −0.35), anti-CD21 therapy (SMD= −0.89 AU/ml, 95%CI: −1.71 to −0.07), anti-CD22 therapy (SMD= −1.38 AU/ml, 95%CI: −2.22 to −0.53), infliximab (SMD= −1.29 AU/ml, 95%CI: −1.81 to −0.76), methotrexate (SMD= −0.82 AU/ml, 95%CI: −1.03 to −0.61), methotrexate monotherapy (SMD= −0.71 AU/ml, 95%CI: −0.97 to −0.44), non-TNF-α (SMD= −0.25 AU/ml, 95%CI: −0.40 to −0.09), ocrelizumab (SMD= −3.25 AU/ml, 95%CI: −3.85 to −2.65), and ustekinumab (SMD= −0.57, 95%CI: −1.12 to −0.02), as well as those without treatment (SMD= −0.17, 95%CI: −0.29 to −0.04). However, data related to other therapies, such as immunomodulators (SMD = 0.15 AU/ml, 95%CI: −0.38 to 0.68), Janus kinase (JAK) inhibitor (SMD = 0.59 AU/ml, 95%CI: −0.42 to 1.59), prednisone monotherapy (SMD= −0.24 AU/ml, 95%CI: −0.64 to 0.16), and vedolizumab (SMD= −0.57 AU/ml, 95%CI: −1.19 to 0.04) showed nonsignificant differences between the two groups (Table 4). Other IgG titer results are described in BAU/ml with nine treatments for markedly significant differences between patients who are immunocompromised and the healthy group (bDMARDs/tsDMARDs: SMD= −0.67, 95%CI: −0.93 to −0.42; csDMARDs: SMD= −0.55, 95%CI: −1.09 to −0.02; fingolimod: SMD= −1.51, 95%CI: −1.79 to −1.23; infliximab: SMD= −0.54, 95%CI: −0.90 to −0.16; JAK inhibitor: SMD= −1.82, 95%CI: −2.33 to −1.32; ocrelizumab: SMD= −1.43, 95%CI: −1.69 to −1.18; rituximab: SMD= −2.33, 95%CI: −2.91 to −1.74; TNF inhibitor: SMD= −0.88, 95%CI: −1.15 to −0.60), but not for methotrexate (SMD= −0.58, 95%CI: −1.24 to 0.08) and no treatment (SMD= −0.25, 95%CI: −0.54 to 0.05). Other data on IgG titer level in the subgroup analysis by treatment is presented in Table 4. The provided data could provide clinical workers and populations knowledge on medical therapies associated with the effectiveness of SARS-CoV-2 vaccines, and help in clinical decision making (Table 5).

**Table 5.** Result of subgroup analysis about NAb inhibitor titer by potential factors.

Subgroup analysis	Item	NAb inhibitor titer
Types of disease	AIDs	−0.66 (−1.57, 0.24)
	ARD	−0.25 (−0.34, −0.15)
	CID	−0.61 (−1.11, −0.11)
	IBD	−0.79 (−1.30, −0.29)
	PAPS	−0.02 (−0.31, 0.26)
	pSS	−0.41 (−0.67, −0.14)
	RA	−0.62 (−1.12, −0.12)
	RD	−1.16 (−1.58, −0.74)
	SLE	−0.11 (−0.36, 0.14)
	SSc	0.23 (−0.44, 0.90)
Treatment	TNFi	0.44 (0.20, 0.69
Immunoassay	ELISA	−0.40 (−0.57, −0.23)
	NR	−0.78 (−1.15, −0.40)
History of SARS-CoV-2 infection	With the history of infection	−0.02 (−0.31, 0.26)
	Without the history of infection	−0.48 (−0.72, −0.24)
	Unknow	−0.42 (−0.71, −0.12)
Vaccine dose	1st dose	−0.40 (−0.72, −0.07)
	2nd dose	−0.44 (−0.63, −0.25)
Vaccine type	Inactivated virus vaccine	−0.29 (−0.43, −0.14)
	mRNA vaccine	−0.76 (−1.25, −0.28)
Vaccine subtype	BBV152	−0.78 (−1.15, −0.40)
	Pfizer	−0.36 (−0.72, 0.01)
	Pfizer and Moderna	−1.34 (−2.62, −0.06)
	Sinovac-CoronaVac	−0.25 (−0.40, −0.11)
Variant of concerns	None	−0.42 (−0.59, −0.25)

*Note:* NAb, neutralising antibody; mRNA, messenger RNA; TNFi, tumour necrosis factor inhibitors; pSS, Primary Sjögren’s syndrome; PAPS, primary antiphospholipid syndrome; CID, chronic inflammatory diseases; AIDs, autoinflammatory diseases; ARD, autoimmune rheumatic diseases; RA, Rheumatoid arthritis; RD, rheumatic disease; SLE, systemic lupus erythematosus; IBD, inflammatory bowel disease; SSc, systemic sclerosis; ELISA, Enzyme-linked immunosorbent assay.

### Subgroup analysis of patients who are immunocompromised by SARS-CoV-2 vaccine dose

The pooled serological response after the first (RR = 0.73, 95%CI: 0.67 to 0.79), second (RR = 0.92, 95%CI: 0.89 to 0.96), and third vaccine doses (RR = 0.50, 95%CI: 0.27 to 0.93) showed reduced prevalence in patients who are immunocompromised compared to the healthy group. In three studies, with a total of 285 patients who are immunocompromised and 153 immunocompetent people, antibody response detected by immunoassay were high among the healthy group (RR = 0.60, 95%CI: 0.49 to 0.73) following the first vaccine dose as well as in the second dose for 16 studies (RR = 0.93, 95%CI: 0.89 to 0.97), whereas only one study reported a high response after the third dose (RR = 0.85, 95%CI: 0.54 to 1.34). In addition, a subgroup analysis of the incidence of seropositive IgG was also conducted and presented very similar results with antibody response, for more data with various vaccine dose (1st dose for RR = 0.73, 95%CI: 0.66 to 0.79; 2nd dose for RR = 0.87, 95%CI: 0.83 to 0.90; and 3rd dose for RR = 0.84, 95%CI: 0.59 to 1.19). Data on the incidence of serological response, antibody response, and seropositive IgG are summarized in Table 3. Interestingly, this meta-analysis also evaluated effectiveness *via* collecting the incidence of NAb and found results consistent with other qualitative data, which represents immunocompetent people could develop sufficient antibodies against the SARS-CoV-2 virus, whereas patients who are immunocompromised could not. Patients had a breakthrough virus infection following the 2nd and 3rd vaccine doses during subsequent virus attacks, although this was not significantly different with healthy group, with RR of 1.28 and 0.94, respectively (fore, 95%CI: 0.99 to 1.66 and later, 95%CI: 0.70 to 1.26). Further subgroup analysis of quantitative data such as antibody and IgG titer level, with abundant data of various units by vaccine dose, demonstrated showed weak antibody titer after the 2nd (SMD= −1.14, 95%CI: −1.27 to −1.01) and 3rd vaccine doses (SMD= −2.84, 95%CI: −3.58 to −2.10) for AU/ml. For BAU/ml which was commonly used across studies, two studies with four data reported a significant difference in antibody titer level between patients who are immunocompromised and the healthy group (SMD= −0.64, 95%CI: −0.86 to −0.42). This finding is strongly supported by IgG titer level in BAU/ml, demonstrating that patients who are immunocompromised failed to develop similar titer level after immunization (1st dose, SMD= −1.46, 95%CI: −1.65 to −1.28 and 2nd dose, SMD= −0.80, 95%CI: −0.90 to −0.70). Studies using other units of measurement also indicated identical results (2nd dose, SMD= −0.72, 95%CI: −1.12 to −0.31 in relative units (RU)/ml; 1st dose, SMD= −0.83, 95%CI: −1.07 to −0.60 and 2nd dose, SMD= −1.14, 95%CI: −1.39 to −0.90 in UA/ml; 2nd dose, SMD= −1.14, 95%CI: −1.39 to −0.90 as U/ml). Only one study by Shenoy et al., with a total of 102 patients with AIRD and non-AIRD controls reported no marked difference between healthy participants and patients who are immunocompromised without describing the unit of measurement.

### Subgroup analysis of patients who are immunocompromised by SARS-CoV-2 vaccine type

Four vaccine types were considered in this meta-analysis. A significant difference in seroconversion rate was not observed in those vaccinated with inactivated virus and adenoviral vector vaccines (RR = 0.93, 95%CI: 0.85 to 1.01). However, patients who are immunocompromised with homologous vaccine strategy (including adenoviral vector vaccine, inactivated virus vaccine, and mRNA vaccine) or combined strategy of mRNA and adenoviral vector vaccine were presented remarkable difference in serological response comparing with healthy participants (RR = 0.81, 95%CI: 0.70 to 0.94, RR = 0.71, 95%CI: 0.65 to 0.78, RR = 0.76, 95%CI: 0.68 to 0.86, and RR = 0.81, 95%CI: 0.72 to 0.92, respectively). The subgroup analysis by vaccine type in antibody response revealed that patients primed with a combined schedule of adenoviral vector vaccine and inactivated virus vaccine (RR = 1.01, 95%CI: 0.89 to 1.15) or homologous administration of the abovementioned two vaccines (adenoviral vector vaccine, RR = 0.75, 95%CI: 0.39 to 1.41; inactivated vaccine, RR = 0.95, 95%CI: 0.91 to 1.00) could generate nonsignificant response rate with the healthy group as the control. Inconsistent results were observed in the incidence of seropositive IgG for patients primed with mRNA and vector vaccines; no significant difference was observed between patients who are immunocompromised and the healthy group (RR = 0.78, 95%CI: 0.59 to 1.02). Moreover, no significant difference in three SARS-CoV-2 vaccines (adenoviral vector vaccine, RR = 0.89, 95%CI: 0.84 to 0.95; inactivated vaccine, RR = 0.70, 95%CI: 0.62 to 0.80; mRNA vaccine, RR = 0.72, 95%CI: 0.65 to 0.80) were observed. Regarding the incidence of seropositive NAb, significant differences in homologous inactivated vaccine or mRNA vaccines were observed between patients who are immunocompromised and the healthy group (RR = 0.79, 95%CI: 0.67 to 0.94; RR = 0.63, 95%CI: 0.54 to 0.74, respectively). Significant differences in IgG titer post-immunization for each unit including AU/ml (SMD for mRNA vaccine= −0.53, 95%CI: −0.62 to −0.44; SMD for inactivated virus vaccine= −1.96, 95%CI: −2.06 to −1.85, SMD for mRNA and adenoviral vector vaccines= −0.77, 95%CI: −0.86 to −0.67), UA/ml (SMD for inactivated virus vaccine= −0.98, 95%CI: −1.15 to −0.81), U/ml (SMD for mRNA vaccine= −1.20, 95%CI: −1.49 to −0.92), and RU/ml (SMD= −0.72, 95%CI: −1.12 to −0.31) were also observed. In addition, antibody titer level demonstrated distinct difference among unit of AU/ml. Specifically, vaccine strategies involving the use of mRNA vaccine also demonstrated statistically significant differences (SMD for single mRNA vaccine= −1.27, 95%CI: −2.12 to −0.43; SMD for mRNA and adenoviral vector vaccine= −2.67, 95%CI: −2.92 to −2.43), but not inactivated virus vaccine (SMD= −0.19, 95%CI: −1.18 to −0.80) for non-unit. Additionally, statistically significant differences in NAb inhibitor titer among inactivated virus vaccine and mRNA vaccine recipients were observed between groups (SMD= −0.29, 95%CI: −0.43 to −0.14 and SMD= −0.76, 95%CI: −1.25 to −0.28, respectively) (Table 5). This meta-analysis also further analyzed vaccine subtypes owing to vaccine brands potentially having different efficacies in people who are immunocompromised (Tables 3 and 4).

### Subgroup analysis of patients who are immunocompromised by SARS-CoV-2 vaccine subtype

As several people worldwide received many vaccine brands that could be biased by confounders, subgroup analyses were performed. For serological response, a combined schedule of Pfizer, Moderna, and Janssen reported by Alexander et al. and Haider et al. showed nonsignificant difference between patients who are immunocompromised and the healthy group (RR = 0.96, 95%CI: 0.86 to 1.08), strategy of BBV152 and AstraZeneca by Shenoy et al. (RR = 0.96, 95%CI: 0.72 to 1.28) and vector-based vaccine by Bsteh et al.: RR = 0.89, 95%CI: 0.67 to 1.19) as well. Meanwhile, most vaccine schedules presented a significantly more favorable seroconversion rate after SARS-CoV-2 vaccination in the healthy population than in patients who are immunocompromised (strategy of Pfizer, Moderna, AstraZeneca, and Janssen: RR = 0.87, 95%CI: 0.82 to 0.93; strategy of Pfizer and Moderna: RR = 0.91, 95%CI 0.85 to 0.98; and single Sinovac-CoronaVac: RR = 0.80, 95%CI: 0.74 to 0.87). A vaccine schedule of Pfizer and Moderna and homologous Pfizer vaccine demonstrated significant difference in antibody response as reported in six studies (RR = 0.61, 95%CI: 0.38 to 0.96). Nevertheless, the homologous vaccine strategy of AstraZeneca (RR = 0.75, 95%CI: 0.39 to 1.41) and mixed vaccine schedules of Pfizer, Moderna, AstraZeneca, and Janssen (RR = 0.93, 95%CI: 0.82 to 1.05); BBV152 and AstraZeneca (RR = 1.01, 95%CI: 0.89 to 1.15); and Pfizer, Moderna, AstraZeneca (RR = 0.60, 95%CI: 0.16 to 2.26) showed no significant difference in antibody response between the two groups. A total of 19 studies provided data on only Pfizer and significant difference in the incidence of seropositive IgG were observed between patients who are immunocompromised and the healthy group (RR = 0.72, 95%CI: 0.65 to 0.80). A similar difference in the vaccine schedule primed with Sinovac-CoronaVac for detectable IgG antibody level by pooling the results of nine studies (RR = 0.70, 95%CI: 0.62 to 0.80). The mixed strategy involving two vaccines, namely Pfizer and Moderna (RR = 0.88, 95%CI: 0.68 to 1.14), Pfizer and AstraZeneca (RR = 0.76, 95%CI: 0.53 to 1.08), and Pfizer, Moderna, AstraZeneca, and Janssen (RR = 0.80, 95%CI: 0.49 to 1.30) yielded varying incidence rates for seropositive neutralizing antibodies (NAb). Only the Sinovac-CoronaVac vaccine demonstrated a significant difference (RR = 0.73, 95%CI: 0.67 to 0.79), compared to Pfizer (RR = 0.92, 95%CI: 0.78 to 1.09) and the mixed strategy of Pfizer and Moderna (RR = 0.80, 95%CI: 0.61 to 1.05). For breakthrough SARS-CoV-2 infections, no significant difference in each vaccine subtype (mixed strategy of Pfizer, Moderna, AstraZeneca, and Janssen: RR = 0.97, 95%CI: 0.72 to 1.30; combined strategy of Pfizer and Moderna: RR = 1.30, 95%CI: 0.83 to 2.03; and Pfizer: RR = 1.26 95%CI: 0.91 to 1.73 and Sinovac-CoronaVac vaccine: RR = 1.96, 95%CI: 0.28 to 13.54) was observed between the two groups. The antibody titer and IgG titer level were lower in patients who are immunocompromised than in the healthy group, with the majority of vaccine strategies demonstrating significant differences, except antibody titer level for those who received Pfizer and Moderna by AU/ml (SMD= −0.19, 95%CI: −0.78 to 0.39), mRNA vaccine by AU/ml (SMD= −0.02, 95%CI: −0.31 to 0.26), BBV152 (SMD= −0.19, 95%CI: −1.18 to 0.80), and AstraZeneca by U/ml (SMD= −0.30, 95%CI: −1.07 to 0.46). Detailed results of the subgroup analysis are presented in Tables 3 and 4. NAb inhibitor titer level by vaccine subtype were analyzed in detail, and the results indicated no statistically significant difference between Pfizer recipients with autoimmune diseases and the healthy group (SMD= −0.36, 95%CI: −0.72 to 0.01). Nevertheless, only one study has reported that the BBV152 vaccine significantly reduces NAb inhibitor titer level in patients with autoimmune diseases (SMD= −0.78, 95%CI: −1.15 to −0.40), whereas several studies have reported the same result for Sinovac-CoronaVac and mixed schedule of Pfizer (SMD= −0.25, 95%CI: −0.40 to −0.11) and Moderna (SMD= −1.34 95%CI: −2.62 to −0.06) (Table 5).

### Subgroup analysis of patients who are immunocompromised by history of SARS-CoV-2 infection

Because many studies have not reported on the history of SARS-CoV-2 infection, the present study collected and analyzed existing data and noted that patients with a history of viral infection had lower response rates of seroconversion (RR = 0.79, 95%CI: 0.70 to 0.89) and antibody response (RR = 0.62, 95%CI: 0.47 to 0.84) following vaccination than those without a viral infection history. However, history of previous infection in terms of incidence of seropositive IgG (RR = 0.87, 95%CI: 0.76 to 1.00) and NAb (RR= 0.91, 95%CI: 0.75, 1.09) was not significantly different between the two groups. Statistically significant differences in serological response (RR = 0.82, 95%CI: 0.75 to 0.90), incidence of seropositive IgG (RR = 0.71, 95%CI: 0.63 to 0.80), and seropositive NAb (RR = 0.68, 95%CI: 0.57 to 0.81) were observed in patients who are immunocompromised without a history of infection. The rest of the results on unknown previous history of SARS-CoV-2 infection with significant differences involved dichotomous data (the incidence of serological response, antibody response, seropositive IgG, and Nab). The subgroup analysis results are summarized in Table 3. Several studies have not described whether the participants recruited in their cohort had prior history of SARS-CoV-2 infection, who were classified as the unknown subgroup based on various units of measurement relating to antibody titer level. The findings associated with subgroup of unknown prior medical history showed significant differences in any unit of IgG titer between the two groups (SMD= −0.69 AU/ml, 95%CI: −0.79 to −0.59; pooling seven studies for SMD= −0.95 BAU/ml, 95%CI: −1.07 to −0.84; one study for SMD= −1.82 OD, 95%CI: −2.63 to −1.02, SMD= −0.82U/ml, 95%CI: −1.21 to −0.43, and SMD= −0.72 RU/ml, 95%CI: −1.12 to −0.31). Additionally, the results from IgG titer with or without medical information history on previous SARS-CoV-2 infection demonstrated significant difference and concordantly favored the healthy group among all units in the included studies (with history of SARS-CoV-2 infection: SMD= −0.68 AU/ml, 95%CI: −0.77 to −0.60; SMD= −0.44 BAU/ml, 95%CI: −0.72 to −0.15; SMD = 0.84 OD, 95%CI: −1.06 to −0.62; SMD= −1.62 U/ml 95%CI: −2.03 to −1.21; without history of SARS-CoV-2 infection: SMD= −1.88 AU/ml, 95%CI: −1.99 to −1.77; SMD= −1.10 BAU/ml, 95%CI: −1.26 to −0.94; SMD= −1.03 OD, 95%CI: −1.36 to −0.70; SMD= −0.98 UA/ml, 95%CI: −1.15 to −0.81). Considering antibody titer level, which differed across situations that participants had during the research period, history of SARS-CoV-2 infection was not significantly different between patients who are immunocompromised and the healthy group (SMD = 0.11 AU/ml, 95%CI: −were classified as the unknown subgroup based on various units of he remaining groups related to antibody titer regardless of history of infection (Table 4). Further analysis of NAb inhibitor titer level reported by Signorelli et al. with first and second doses, presented nonsignificant difference in patients with a history of SARS-CoV-2 infection (SMD= −0.02, 95%CI: −0.31 to 0.26), whereas reduced NAb inhibitor titer was observed in patients who are immunocompromised without a history of SARS-CoV-2 infection (SMD= −0.31 95%CI: −0.39 to −0.22) and those with unknown medical coronavirus infections (SMD= −0.42, 95%CI: −0.71 to −0.12) (Table 5).

### Subgroup analysis of patients who are immunocompromised by SARS-CoV-2 variant of concern

Variants of concerns were considered into this meta-analysis. Few studies have reported the Delta (B.1.617.2) and B.1.1.7 variants as critical factors triggering vaccine immunogenicity against the SARS-CoV-2 virus. Nonetheless, a significantly remarkable difference was observed in any condition of variants of concern and even in those of unknown periods. Thus, reduced effectiveness was observed against the Delta and B.1.1.7 variants in patients who are immunocompromised compared to that in the healthy group (Tables 3 and 4).

Patients who are immunocompromised and received SARS-CoV-2 vaccines against the Delta (B.1.617.2) variant had significantly lower IgG titer (SMD= −1.02 AU/ml, 95%CI: −1.18 to −0.86) and antibody titer level than the healthy group (SMD= −2.67, 95%CI: −2.92 to −2.43).

### Subgroup analysis of patients who are immunocompromised by SARS-CoV-2 immunoassay

Immunoassay after immunization was analyzed using various assays, such as enzyme-linked immunosorbent assay (ELISA) and indirect chemiluminescence immunoassay (CLIA), that laboratory diagnosis to SARS-CoV-2 infection, might have influence on detective seropositive antibody rate. Hence, this subgroup analysis of serological response illustrated that most studies used immunoassays or kits were ELISA (RR = 0.86, 95%CI: 0.79 to 0.94) or CLIA (RR = 0.89, 95%CI: 0.84 to 0.95), and showed significant differences between the two groups, except for the combined ELISA and CLIA method (RR = 0.99, 95%CI: 0.86 to 1.14). Beck Coulter by Haider et al. also revealed nonsignificant difference for seroconversion rate between patients who are immunocompromised and the healthy group (RR = 0.92, 95%CI: 0.79 to 1.07). Using electrochemiluminescence immunoassay analyzer (ECLIA) or CLIA as measure tool for detecting antibody response in their bodies showed a significant difference between patients who are immunocompromised and the healthy group (RR = 0.83 95%CI: 0.74 to 0.93, RR = 0.83 95%CI: 0.74 to 0.93, respectively). The study by Verstappen et al. adopted indirect CLIA and luminescent immunoassay microparticles (MIA) analyzed involved patients for antibody response illustrated statistical difference between two groups (RR = 1.00, 95%CI: 0.74 to 1.34). Immunoassay tools including ELISA, CLIA, and chemiluminescent immunoassay microparticles (CMIA) used to detect seropositive IgG against coronavirus demonstrated significant differences between the two groups (RR = 0.73, 95%CI: 0.64 to 0.83; RR = 0.69, 95%CI: 0.61 to 0.78; RR = 0.82, 95%CI: 0.65 to 1.03, respectively), except for ECLIA in four studies (RR = 0.92, 95%CI: 0.86 to 0.99). NAb incidence was significantly different with ELISA (RR = 0.70, 95%CI: 0.60 to 0.82) and CLIA (RR = 0.32, 95%CI: 0.17, 0.60) tools between patients who are immunocompromised and the healthy group. Many studies adopting the CLIA method for measuring antibody titer post-vaccination showed significant differences on various units (AU/ml: SMD= −2.26, 95%CI: −2.43 to −2.08; BAU/ml: SMD= −1.63, 95%CI: −1.78 to −1.47; U/ml: SMD= −0.45, 95%CI: −0.70 to −0.20). The inconsistent results in antibody titer were revealed using ELICA for different units. In particular, no statistically significant difference was observed when using the unit BAU/ml (SMD= −0.02, 95%CI: −0.31 to 0.26), compared to ELICA in U/ml (SMD= −1.06, 95%CI: −1.25 to −0.86) and mg/ml (SMD= −6.08, 95%CI: −6.78 to −5.38) between the two groups. Only one study by Duengelhoef et al. published in 2020 reported contradictory results when using the mixed method of two units of measurement, demonstrating significant differences in BAU/ml (SMD= −7.36, 95%CI: −13.13 to −1.59) and nonsignificant difference in AU/ml (SMD = 0.11, 95%CI: −0.09 to 0.30). Ten studies used the CLIA tool to detect IgG titer, and showed favorable result between the two groups (SMD= −1.07 AU/ml, 95%CI: −1.89 to −0.24). Two studies with eight available data used the CLIA and ELISA tools throughout the first and second vaccine doses in patients on TNF treatment (SMD= −0.65 AU/ml, 95%CI: −1.25 to −0.05). The ECLIA method was used for participants who received CoronaVac or BioNTech vaccines, and nonsignificant difference was noted between the two groups (SMD= −0.13 AU/ml, 95%CI: −0.35 to 0.09). However, some studies used ELISA for IgG titer, not ECLIA, thus yielding a significant difference between the two groups (SMD= −0.78 AU/ml, 95%CI: −1.03 to −0.54). In addition, serval immunoassays used BAU/ml to detect IgG titer following vaccines which presented significant difference (CLIA in seven studies: SMD= −1.29, 95%CI: −1.65 to −0.93; ELISA in two studies: SMD= −0.54 95%CI: −0.84 to −0.24) (Tables 3 and 4**)**. Two studies utilized the ECLIA tool for IgG titer with the U/ml (SMD= −1.27, 95%CI: −1.55 to −0.98) and CLIA for UA/ml (SMD= −0.99, 95%CI: −1.29 to −0.68), with both reporting significant differences between the two groups. Furthermore, almost all of studies adopted the ELSA method to detect NAb inhibitor titer against SARS-CoV-2 virus post-immunization, which presented statistically significant difference between the two groups in a pooled result of SMD −0.40 with 95%CI of −0.57 to −0.23 (Table 5).

## Discussion

This large systematic review was designed to identify an optimal immunization schedule by comparing the effectiveness of SARS-CoV-2 vaccines between patients who are immunocompromised and a healthy group in terms of vaccine dose, brand or subtype, therapeutic schedule, and history of SARS-CoV-2 infection.

Majority of the included studies were conducted in the United States, Germany, United Kingdom, Sweden, and Israel. Only a few studies have described the certain ethnicity of their participants. Moreover, the population comprised adults (age > 18 years) who were either healthy or had autoimmune diseases.

Patients diagnosed with autoimmune diseases showed reduced seroconversion after vaccination compared to healthy participants, which accords with results from a previous network meta-analysis [10]. Exclusive reliance on seroconversion as single criteria to assess humoral response may result in false negative serological results. Thus, this meta-analysis collected data on seropositive neutralizing antibodies from appropriate studies and reached a similar conclusion for IgG and antibody response.

Continuous data such as IgG titer and Nab inhibitor titer level were used to assess the immunity of patients with autoimmune diseases who received SARS-CoV-2 vaccines. The results revealed lower levels in patients who are immunosuppressed than the healthy group. In addition, only breakthrough infection after immunization had no significant difference between the two groups, and few studies have reported on the emergency of new variants of the virus, such as the Delta (B.1.617.2), variant and disease severity [101]. Therefore, further subgroup analyses were conducted to evaluate an optimal vaccine schedule for patients with autoimmune diseases, based on the types of autoimmune disease, administration of specific treatment on autoimmune diseases, vaccine dose, vaccine types or subtypes, immunoassay, variants of concern, and history of prior SARS-CoV-2 infection.

Favorable results were observed on seroconversion rate for patients who are immunocompromised with IBD and IMDs; antibody response rate in patients with AID, IBD, neuroimmunologic disease and MS; and incidence of positive IgG in patients with RD, Behçet’s syndrome (BS), CVID, IBD, psoriasis, primary Sjögren’s syndrome (pSS) and systemic sclerosis (SSc). Patients with IBD demonstrated a comparable antibody response with healthy controls. Therefore, patients who are immunocompromised should receive vaccination according to their own disease conditions. Furthermore, the potential influence of immunosuppressants that patients take during SARS-CoV-2 vaccination has not been completely explored yet. Patients taking ustekinumab, infliximab, vedolizumab, tofacitinib, prednisone, hydroxychloroquine, TNF inhibitor monotherapy, methotrexate, anti-TNF and methotrexate, sulfasalazine, leflunomide, and adalimumab developed comparable serological response with the healthy population. In addition, the subgroup analysis revealed that patients on ustekinumab or withheld DMARD therapy had similar antibody titer level with the healthy control. Most drugs administered for autoimmune diseases reduced IgG titer level, as evidenced by the lower IgG titer level in patients who are immunocompromised than in healthy controls, following SARS-CoV-2 vaccination. The subgroup analysis of the history of SARS-CoV-2 infection revealed unfavorable IgG titer level in patients who are immunocompromised, regardless of history of infections, compared to that in healthy controls. Additionally, variants of concern was also identified as an influential factor and presented similar results as the history of SARS-CoV-2 infection, which might be attributed to limited relevant research. In addition, the factors of SARS-CoV-2 vaccines have been considered and analyzed further in terms of dose, type, and subtype. Most results in the subgroup analysis of vaccine dose, revealed weak evidence that patients who are immunocompromised failed to develop sufficient seropositive antibody rate or seroconversion rate compared to healthy controls. However, although the incidence of seropositive IgG with a third vaccine dose showed a different trend, three vaccine doses are necessary for patients with immunodeficiency. However, the optimal strategy for patients who are immunocompromised to develop antibodies for viral invasion close to that of the healthy population who received SARS-CoV-2 vaccines remains unclear. Moreover, this meta-analysis used mainstream vaccines subtype and type, and identified subgroups of inactivated virus and adenoviral vector vaccines in patients who achieved similar titer and seropositive antibody rate with those in the healthy population. Detailed subgroup analysis of vaccine subtype, technically, brands of those in lieu of types, revealed complex results in combined vaccine strategies such as Pfizer, Moderna, AstraZeneca, and Janssen; Pfizer and AstraZeneca; BBV152 and AstraZeneca; and Pfizer and Moderna. Altogether, the results indicate that patients who are immunocompromised receiving multiple vaccine brands or subtypes might generate a similar level of specific antibody and antibody titer as that of the healthy population. However, the main determinant of cell entry and tropism called spike (S) protein of SARS-CoV-2, contains neutralizing epitopes triggering NAb in bodies, was explored by various approaches and techniques throughout many original studies to develop candidate vaccines against coronavirus. Therefore, NAb and its inhibitor titer were also analyzed in this study. Our findings emphasize that patients diagnosed with IBD, pSS, PAPS, and SSc; on vedolizumab, methotrexate, ustekinumab, and infliximab therapy; and with previous history of SARS-CoV-2 infections have similar detected titer level or incidence of seropositive NAb inhibitor with healthy controls.

This systematic review and meta-analysis has several strengths. First, this study analyzed some diseases that previous meta-analysis did not [11]. We also further analyzed more detailed patient characteristics including various diseases and treatments, from multidimensional outcomes included in the subgroup analyses. Second, we included SARS-CoV-2 vaccine platforms without any limit of dose and timepoint, which aligns with the meta-analysis of Widhani et al. [12], wherein eligible studies are non-randomized controlled trials. Regarding immunogenicity results, as patients had lower total antibody level than healthy controls, this was well-matched in this study. Third, this study underscores the strong need of administering SARS-CoV-2 vaccines to patients with autoimmune diseases to face current and future epidemics. This study also provides valuable data for healthcare workers in guiding patients with autoimmune decisions regarding clinical treatment decisions and protective measures.

## Limitations

First, studies exploring homologous diseases or treatment on SARS-CoV-2 vaccines for patients who are immunocompromised are limited. Second, few studies have described the history of SARS-CoV-2 infection of enrolled participants, which may have biased the results. Lastly, the cuff-off value associated with the incidence of seropositive IgG or NAb, even serological response, is not consistent among included studies. Hence, false negative results may have been missed, and more researchers used various measure tools to avoid missing truly included patients in their own studies.

## Conclusions

Patients with autoimmune diseases who received most vaccines elicited poorer humoral responses and seropositive incidence after receiving SARS-CoV-2 vaccines than healthy individuals. Despite no observable favorable results of patients who are immunocompromised with autoimmune diseases occurred compared to healthy populations, which has trend of effectiveness of SARS-CoV-2 vaccines close to that of the healthy population. A more precise and detailed schedule of SARS-CoV-2 vaccination considering vaccine subtypes, dose(s), variants of concern (epidemic background of patients involved), and immunoassay used (tools or methods) is needed for patients with autoimmune diseases.

## Supplementary Material

PRISMA checklist.docx

## Data Availability

The data are available from the corresponding author upon reasonable request.
